# Phylogeography and Ecological Niche Modeling Reveal Reduced Genetic Diversity and Colonization Patterns of Skunk Cabbage (*Symplocarpus foetidus*; Araceae) From Glacial Refugia in Eastern North America

**DOI:** 10.3389/fpls.2018.00648

**Published:** 2018-05-22

**Authors:** Seon-Hee Kim, Myong-Suk Cho, Pan Li, Seung-Chul Kim

**Affiliations:** ^1^Department of Biological Sciences, Sungkyunkwan University, Suwon, South Korea; ^2^Key Laboratory of Conservation Biology for Endangered Wildlife of the Ministry of Education, Laboratory of Systematic and Evolutionary Botany and Biodiversity, College of Life Sciences, Zhejiang University, Hangzhou, China

**Keywords:** Araceae, eastern North America, ecological niche modeling, glaciation cycles, phylogeography, skunk cabbage, *Symplocarpus foetidus*

## Abstract

Alternating glacial and interglacial periods during the Quaternary have dramatically affected the distribution and population genetic structure of plant and animal species throughout the northern hemisphere. Surprisingly, little is known about the post-glacial recolonization history of wetland herbaceous perennials that are widely distributed in the understory of deciduous or mixed deciduous-evergreen forests in eastern North America. In this study, we investigated infraspecific variation among 32 populations of skunk cabbage, *Symplocarpus foetidus*, to test the hypothesis that the extant species diversity of skunk cabbage is the result of a post-glacial range expansion from southern refugia during the Quaternary Ice Age. A total of 4041 base pairs (bp) of the chloroplast intergenic spacer region (cpDNA) was sequenced from 485 individuals sampled from glaciated (18 populations, 275 individuals) and unglaciated (14 populations, 210 individuals) regions east and west of the Appalachian Mountains. Haplotype number, haplotype diversity, and nucleotide diversity were calculated, and genetic variation within and among populations was assessed by analysis of molecular variance (AMOVA). The geographic pattern of genetic differentiation was further investigated with a spatial analysis of molecular variance (SAMOVA). A total of eight haplotypes and three genetic groups (SAMOVA) were recovered and a much higher haplotype number (eight haplotypes) and haplotype diversity (0.7425) was observed in unglaciated compared to glaciated populations (five haplotypes, haplotype diversity = 0.6099). All haplotypes found in glaciated regions represented a subset of haplotypes found in unglaciated regions. Haplotypes of *S. foetidus* likely diverged during the Tertiary (mid-Miocene and late Pliocene), predating the last glacial maximum (LGM). Predictions based on ecological niche modeling (ENM) suggested that there was considerably less suitable habitat for skunk cabbage during the LGM, and the habitat range was further south compared to the current distribution. Reduced variation and a subset of haplotypes in glaciated regions suggest a founder effect associated with range expansion via long-distance seed dispersal. Our results do not support the “Driftless Area” scenario for the northern refugium, rather the data suggest a “Northeastern” refugium near the southernmost extent of the LGM.

## Introduction

During the Quaternary Period, climatic oscillations profoundly affected species distribution as well as population genetic structure across the Northern Hemisphere (Hewitt, 2000, 2003). In particular, temperate deciduous forests in eastern North America are thought to be the remnants of the so-called Arcto-Terriary Geoflora, believed to have been eliminated by severe climate change during the Quaternary (Reid et al., 1935; Chaney, 1944; Davis, 1983a,b). Repeated glaciations shifted species ranges and isolated populations primarily in southern refugia (Davis, 1983b; Bennett et al., 1991; Latham and Ricklefs, 1993; Williams et al., 2000). As the glaciers retreated during interglacial periods, species' range gradually expanded northward, and repeated cycles of glacial contraction and expansion generated a distinct genetic structure, i.e., “southern richness” and “northern purity” (Hewitt, 1996, 1999; Peirson et al., 2013). It has been suggested that several factors, such as the speed and duration of range contractions and range shifts, habitat fragmentation during a range expansion, and dispersal abilities or strategies, have influenced genetic diversity and population genetic structure over the course of climate change cycles (Arenas et al., 2012, 2014; Mona et al., 2014). Phylogeographic patterns as well as palynological and paleontological records in Europe and eastern North America have underscored the importance of southern glacial refugia to many temperate trees and forest understory plants (Davis, 1983a; Taberlet et al., 1998; Hewitt, 2000; Soltis et al., 2006). Recent studies suggest that the upper Midwest's “Driftless Area” played an important role as a northern glacial refugium for a diverse assemblage of organisms (Jaramillo-Correa et al., 2004; Rowe et al., 2004; Godbout et al., 2005; Mclachlan et al., 2005; Li et al., 2013). In Europe, key southern refugia include three main peninsulas - the Iberian, Italian, and Balkan–while there is a more complex pattern of glacial refugia in eastern North America (Taberlet et al., 1998; Hewitt, 1999; Soltis et al., 2006; Schmitt, 2007). In the Pacific Northwest, glacial refugia existed both north and south of the ice sheets during the last glacial maximum (LGM), suggesting that complex range expansion scenarios were responsible for post-glacial migration of species (Soltis et al., 1997; Beatty and Provan, 2010).

Phylogeography aims to relate evolutionary processes to spatial, temporal and environmental factors in an effort to understand past and present biodiversity (Avise et al., 1987; Hickerson et al., 2010). Phylogeography is particularly useful when investigating post-glacial migration and dispersal patterns of herbaceous plants that are not well represented in the palynological record (Cruzan and Templeton, 2000; Hewitt, 2000). While palynological studies have provided some information concerning the post-glacial history of eastern North American plant species, relatively few phylogeographic studies have focused on this region (Davis, 1983a; Sewell et al., 1996; Echt et al., 1998; Maskas and Cruzan, 2000; Walter and Epperson, 2001; Griffin and Barrett, 2004; Li et al., 2013), and most North American phylogeographic studies have targeted woody plants (this is also true for studies in Europe; Taberlet et al., 1998). Recently, molecular markers have been used to investigate herbaceous species whose ranges span both glaciated and unglaciated portions of eastern North America (e.g., Dorken and Barrett, 2004; Griffin and Barrett, 2004; Gonzales et al., 2008; Fehrmann et al., 2012; Li et al., 2013; Barnard-Kubow et al., 2015). Two recent studies that describe similar species distribution patterns as those found in this study (i.e., long-lived herbaceous perennials and widely spread in understory deciduous or mixed deciduous-evergreen forests in eastern North America) are worth pointing out. First, Griffin and Barrett (2004), in a study of *Trillium grandiflorum* (Melanthiaceae) found no significant reduction in genetic diversity in glaciated vs. unglaciated regions. The authors also provide evidence that restricted gene flow shaped population differentiation, and that more pollen flow relative to seed dispersal was important in the occasional long distance dispersal of species during northward migration. Second, Li et al. (2013), while investigating the *Smilax herbacea* complex (Smilacaceae) in the eastern U.S. and southeast Canada, found an Appalachian discontinuity, with multiple refugia both east and west of the Appalachians. The authors stressed the importance of a “Driftless Area,” described as a Midwestern northern refugium distinct from southern refugia in the U.S., and postulated contact between the Midwest and the East Coastal lineages during expansion. Although the two plant species from these relative studies share similar life history traits and distribution (eastern U.S. and southeastern Canada), different patterns of genetic structure and phylogeography were observed. Thus, additional studies are necessary in order to explain emerging patterns of the distribution and genetic structure of herbaceous plant species found in the understory of temperate forests in eastern North America.

Ecological niche modeling (ENM) is a relatively recent innovation that has greatly enhanced phylogeographic studies, in particular those of systems that have few physical specimens such as species that diverged in the Pleistocene refugia (Alvarado-Serrano and Knowles, 2014). ENM is a useful tool for predicting the distribution of species during paleoclimatic reconstructions (Waltari et al., 2007). Along with genetic diversity estimates, refugia hypotheses can be tested using quantitative estimates derived from ENM predications (Chan et al., 2011; Gavin et al., 2014; Barnard-Kubow et al., 2015; Loera et al., 2017; Scheinvar et al., 2017). For example, ENM was used to make parallel predictions for 20 North American terrestrial vertebrate species. In 14 of 20 species (70%), significant spatial correlations were found between the predictions from ENM and predictions based on phylogeographic studies (Waltari et al., 2007). ENM based studies, however, are sensitive to various issues (e.g., theoretical assumptions, model classes, projections in non-analogous climates, etc.), and thus rigorous procedures to hindcast ENMs were recommended (Nogués-Bravo, 2009 and references therein; Alvarado-Serrano and Knowles, 2014).

**Figure 1:**
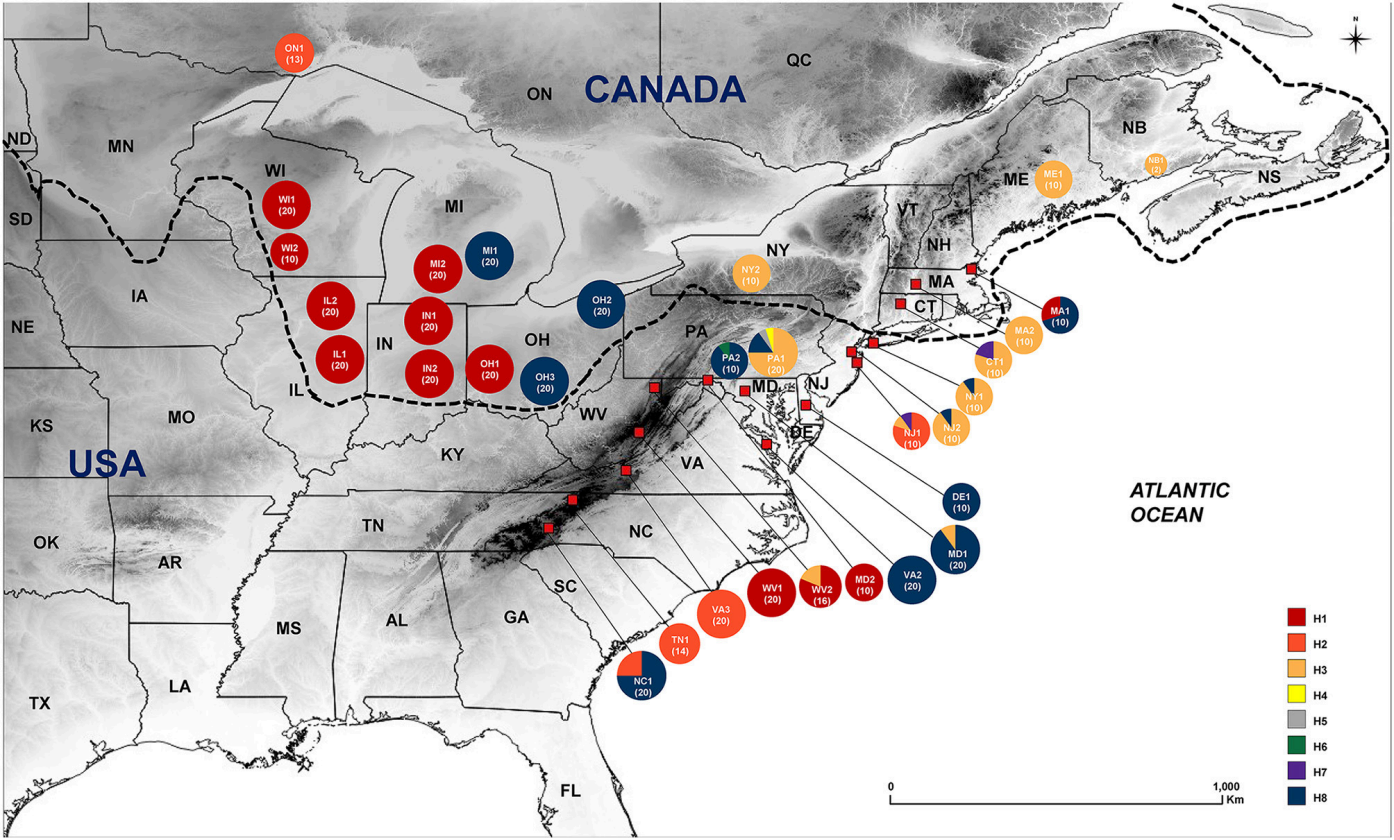
Geographic distribution of eight haplotypes of *Symplocarpus foetidus* across 32 collection sites in eastern North America. Color proportion in each pie chart represent haplotype frequencies. Dotted line indicates approximate southern boundary of Last Glacial Maximum (Mickelson and Colgan, 2003).

Of the five recognized species in the genus *Symplocarpus* (Araceae), only *S. foetidus* (L.) Salisb. ex W. P. C. Barton, is likely native to North America, with a vast distribution in eastern North America. Like other congeneric species in East Asia, *S. foetidus* occurs in swamps, wet woods, along streams, and other wet low areas (0–1,100 m). *S. foetidus* is common in southeastern Canada (Ontario, Quebec, New Brunswick, and Nova Scotia) and the eastern U.S., ranging from Minnesota to North Carolina and Tennessee. However, at its southern range limit, *S. foetidus* is less common and is an endangered species in Tennessee, being highly restricted to the northeastern corner of the state (i.e., Johnson County) (Wilson, 1960; Wen et al., 1996; Thompson, 2005; Figure 1). Like its closest relative, *S. renifolius* in East Asia (Nie et al., 2006), *S. foetidus* in eastern North America blooms from late winter through spring before leaves emerge. Seeds usually fall into the muddy substrate or are carried off by animals and floods. Although the range of dispersal of seeds is unknown in *S. foetidus*, the mean dispersal distance of its sister species, *S. renifolius* (in Japan) has been estimated at approximately 10 m, likely facilitated by rodents (Wada and Uemura, 1994). Thus, rodents are likely important seed dispersers for plants in this genus in East Asia. Dispersal of *S. foetidus* by larger animals such as large mammals and birds, however, has been suggested in eastern North America, and germination has been documented following ingestion of *S. foetidus* seeds by various vertebrate species (Knutson, 1979). Of note is the spring feeding of black bears (*Ursus americanus*) in North America, where half of their diet in 1994 and almost the entire diet (99%) in 1996 (Iverson et al., 2001) was *S. foetidus*. The remarkably large range of American black bears (up to 200 km, Rogers, 1987; maximum 168 km, Noyce and Garshelis, 2011) suggests that these organisms could play a role in the long-range dispersal of *S. foetidus*. Long-range seed dispersal by such animals in eastern North America could result in extensive gene flow, and thus little genetic variation and population structure are expected. In contrast, restricted gene flow, spatial demography, and population differentiation are expected for plants dispersed by scatter-hoarding animals (Vander Wall and Beck, 2012).

*Symplocarpus foetidus* is an ideal model system with which to examine the areas of southern and northern refugia, northward migration routes and the genetic structure between glaciated and ice-free regions during the Quaternary, as it is a common understory herb in swamps and wet woods in both glaciated and unglaciated parts of eastern North America. This study represents the first analysis of the population genetic structure and phylogeographic patterns of *S. foetidus*. Here, we sampled populations of *S. foetidus* and sequenced four chloroplast DNA (cpDNA) noncoding regions to elucidate the refugial and recolonization history of herbaceous *S. foetidus* in eastern North America. Specifically, the aim of this study was to address the following questions: (1) Is there evidence of reduced genetic diversity in recolonized populations in the northern range limit? We would expect a trend of south to north decreasing genetic diversity as a result of repeated founder effect if the current populations of *S. foetidus* arose from a southern glacial refugium. (2) Is there evidence for the existence of the Driftless Area northern refugium for *S. foetidus*? If this area has served as a suitable refugium during the LGM, we expect higher genetic diversity, increased relative abundance of endemic and ancestral alleles, and genetic divergence among refugia. In addition, sharing of some unique/ancestral haplotypes between the Driftless Area and the glaciated areas of the Midwest is expected. Finally, (3) are there post-glacial colonization patterns in the present geographical range of *S. foetidus*?

## Materials and methods

### Plant materials

A total of 485 *S. foetidus* individuals, representing 32 populations in the U.S. and Canada, were sampled (Table 1). The number of sampled individuals per population ranged from two (NB1, Canada) to 20 individuals, with an average of approximately 15 individuals per population. Our samples represent most of the range of *S. foetidus*, from the northernmost population (Ontario; approximately 45° latitude) to the southernmost population (North Carolina; approximately 35° latitude). The approximate extent of the ice sheets at the Last Glacial Maximum was based on Dyke et al. (2002) and Mickelson and Colgan (2003). We sampled 14 populations (210 individuals) and 18 populations (275 individuals) from unglaciated and glaciated regions, respectively (Figure 1 and Table 1). For each population, we randomly sampled individuals as far apart as possible (i.e., at least 10 m between individuals). Young, healthy leaves were collected in the field and dried with silica gel for subsequent DNA extraction. Voucher specimens for representative individuals of each population were deposited at Ha Eun Herbarium (SKK) of Sungkyunkwan University. *S. renifolius* from Japan was also sampled to root TCS network construction.

**Table 1:** Populations of *Symplocarpus foetidus* sampled in this study.

**Code**	**Country**	**State**	**Collection Site**	**GPS**	**N/I**	**Haplotypes**
**UNGLACIATED REGIONS (14 POPULATIONS WITH 210 INDIVIDUALS)**						
DE1	USA	Delaware	Felton, Santa Cruz County	38°59′25.4″N 75°32′43.3″W	10	H8 (10)
MD1	USA	Maryland	Frederick County	39°22′05.4″N 77°11′25.1″W	20	H3 (2) H8 (18)
MD2	USA	Maryland	13501-14099 Locker Rd.	39°40′53.6″N 78°12′30.4″W	10	H1 (10)
NC1	USA	North Carolina	Asheville, Buncombe County	35°36′48.3″N 82°34′09.4″W	20	H2 (5) H8 (15)
NJ1	USA	New Jersey	Wall Township, Monmouth County	40°09′40.8″N 74°08′14.6″W	10	H2 (8) H3 (1) H7 (1)
NJ2	USA	New Jersey	Matawan, Monmouth County	40°26′14.6″N 74°16′04.8″W	10	H3 (9) H8 (1)
NY1	USA	New York	Nassau County	40°40′41.8″N 73°41′42.6″W	10	H3 (9) H8 (1)
PA1	USA	Pennsylvania	Harrisburg, Dauphin County	40°18′38.0″N 76°53′00.7″W	20	H3 (15) H4 (1) H5 (1) H8 (3)
PA2	USA	Pennsylvania	Fayetteville, Franklin County	39°54′38.1″N 77°28′23.6″W	10	H6 (1) H8 (9)
TN1	USA	Tennessee	Johnson County	36°23′38.9″N 81°54′32.5″W	14	H2 (14)
VA2	USA	Virginia	Totuskey Creek, Richmond County	37°54′29.9″N 76°35′53.9″W	20	H8 (20)
VA3	USA	Virginia	Blacksburg, Montgomery County	37°11′51.2″N 80°26′47.7″W	20	H2 (20)
WV1	USA	West Virginia	Greenbrier River Trail	38°14′05.0″N 80°05′04.2″W	20	H1 (20)
WV2	USA	West Virginia	King wood old shore camp	39°28′22.9″N 79°41′14.7″W	16	H1 (13) H3 (3)
**GLACIATED REGIONS (18 POPULATIONS WITH 275 INDIVIDUALS)**						
CT1	USA	Connecticut	Hamden, New Haven County	41°45′11.2″N 72°55′31.6″W	10	H3 (8) H7 (2)
IL1	USA	Illinois	61831, Collison	40°14′34.2″N 87°47′01.6″W	20	H1 (20)
IL2	USA	Illinois	Sugar Grove, Kane County	41°47′04.6″N 88°26′29.9″W	20	H1 (20)
IN1	USA	Indiana	Bristol, Elkhart County	41°43′18.9″N 85°45′46.9″W	20	H1 (20)
IN2	USA	Indiana	Anderson, Madison County	40°05′50.4″N 85°37′18.7″W	20	H1 (20)
MA1	USA	Massachusetts	Georgetown, Essex County	42°42′06.6″N 71°00′05.3″W	10	H1 (3) H8 (7)
MA2	USA	Massachusetts	Amherst, Hampshire County	42°18′21.6″N 72°31′47.3″W	10	H3 (10)
ME1	USA	Maine	provided by Chris Campbell (University of Maine)	NA	10	H3 (10)
MI1	USA	Michigan	8754 Coleman Rd.	42°46′21.8″N 84°23′17.6″W	20	H8 (20)
MI2	USA	Michigan	Augusta, Kalamazoo County	42°21′47.0″N 85°21′15.3″W	20	H1 (20)
NY2	USA	New York	Nassau County	42°22′34.0″N 76°52′38.9″W	10	H3 (10)
OH1	USA	Ohio	Troy, Miami County	40°00′58.9″N 84°19′23.3″W	20	H1 (20)
OH2	USA	Ohio	Brukner, Ashtabula County	41°53′23.9″N 80°39′24.1″W	20	H8 (20)
OH3	USA	Ohio	Fairfield County	39°38′19.3″N 82°34′08.6″W	20	H8 (20)
WI1	USA	Wisconsin	2316 Whiting Rd, Stevens Point	44°29′18.0″N 89°34′13.0″W	20	H1 (20)
WI2	USA	Wisconsin	Merrimac, Sauk County	43°24′35.2″N 89°38′12.0″W	10	H1 (10)
NB1	CANADA	New Brunswick	Stock Farm Road	45°26′19.4″N 65°54′08.9″W	2	H3 (2)
ON1	CANADA	Ontario	Thunder Bay	48°24′59.3″N 89°19′48.1″W	13	H2 (13)

*Vouchers were deposited at Ha Eun Herbarium (SKK) of Sungkyunkwan University. N/I, Number of individuals sampled; NA, not available. Haplotype designation was based on Table 2 with individual numbers in parenthesis for each haplotype*.

### DNA extraction, polymerase chain reaction amplification, sequencing, and sequence alignment

Total genomic DNA was extracted from silica gel-dried samples using the DNeasy Plant Mini Kit (Qiagen, Carlsbad, CA, USA), following the manufacturer's instructions. Given the lack of available nuclear genes and simple sequence repeat markers (SSRs), we sequenced plastid DNA to acquire haplotypes for phylogeographic analyses. Twenty-nine noncoding regions (from *rpl*32-*trn*L to *trn*L-*trn*F) based on the normalized PIC (potentially informative characters) values (Figure 4 of Shaw et al., 2007) were screened for variation in populations of *S. foetidus*. Based on an initial survey, four intergenic spacers (*psb*J-*pet*A, *trn*Q-5′*rps*16, *rpl*32-*trn*L, and *trn*S-*trn*G) of the large single copy region were selected for their relatively high level of variation. Then these four regions were used to survey all 485 accessions. The PCR mixture consisted of the template DNA, 0.5 μM of each primer, 1.25 units of Inclone™ Taq polymerase (Inclone Biotech, Yongin, Korea), 100 mM dNTPs, and 2.5 mM MgCl_2_. Polymerase chain reaction (PCR) amplification was done using the following program: initial denaturation at 95°C for 5 min followed by 35 cycles of denaturation at 95°C for 1 min, primer annealing at 54–56°C for 1 min, and primer extension at 72°C for 2 min; followed by a final extension step of 10 min at 72°C. PCR products were stained with Loading STAR staining (DYNE Bio, Seongnam, Korea) on a 1% agarose gel and visualized under ultraviolet light. The amplified PCR products were purified with Inclone^TM^ Gel & PCR purification kit (Inclone Biotech, Yongin, Korea) and subsequently sequenced using a BigDye Terminator v3.1 Cycle Sequencing kit (Applied Biosystems) at Geno Tech Corporation (Daejeon, Korea). The program Sequencher 4.7 program (Gene Codes Corporation, Ann Arbor, MI, USA) was used to assemble the contigs and edit the sequences. Haplotype sequences of *S. foetidus* were deposited in GenBank: accession numbers MH064178-MH064185 for *psb*J-*pet*A, MH064186-MH064193 for *trn*S-*trn*G, MH064194-MH064201 for *rpl*32-*trn*L, and MH064202-MH064209 for *trn*Q-5′*rps*16.

### Data analysis

All cpDNA sequences were aligned with MAFFT version 7 (Katoh and Standley, 2013) using the E-INS-i algorithm and manually adjusted using MacClade 4.04 (Maddison and Maddison, 2002). For maximum likelihood (ML) analysis, non-mononucleotide repeat insertions and deletions (indels) were coded as simple binary characters (Simmons and Ochoterena, 2000) using the SeqState 1.4.1 program (Müller, 2005), while an inversion was manually coded as a single event. The ML analysis was conducted using the K3Pu+I model (Kalyaanamoorthy et al., 2017) of substitution in IQ-TREE version 1.5.5 (Nguyen et al., 2015) under the Bayesian Information Criterion, and branch support was estimated from 1,000 bootstrap replicates (Minh et al., 2013). *S. renifolius* (Akita, Miyagi, and Hokkaido Prefectures, Japan) was used as outgroup based on previous phylogenetic results (Nie et al., 2006). Genealogical relationships among cpDNA haplotypes were inferred from a statistical parsimony network implemented in the program TCS 1.21 (Clement et al., 2000). Missing data and gaps were excluded from the analysis. A haplotype network was also constructed using the median-joining network method (Bandelt et al., 1999) implemented in the software Network 5.0.0.1 (http://www.fluxus-engineering.com). Genetic variation within and among populations was assessed by analysis of molecular variance (AMOVA) implemented in Arlequin 3.5 (Excoffier and Lischer, 2010). General descriptive statistics and estimates of haplotype diversity (Hd; Nei, 1987) and nucleotide diversity (π; Nei, 1987; Tajima, 1989) were estimated with Arlequin 3.5 (Excoffier and Lischer, 2010). The geographic range of *S. foetidus* was partitioned into two geographic regions: glaciated and unglaciated. We did not group populations into east vs. west of the Appalachian Mountains because some northeastern parts of the mountain range were glaciated during the Wisconsin glaciation (glacial extent after Dyke et al., 2002 and Mickelson and Colgan, 2003). Of the 32 populations (485 accessions) sampled in this study, the glaciated region included a total of 18 populations (275 accessions; CT1, IL1, IL2, IN1, IN2, MA1, MA2, ME1, MI1, MI2, NY2, OH1, OH2, OH3, WI1, WI2, NB1, and ON1) and the unglaciated region includes 14 populations (210 accessions; DE1, MD1, MD2, NC1, NJ1, NJ2, NY1, PA1, PA2, TN1, VA2, VA3, WV1, and WV2). NY1 was classified as an unglaciated region owing to its east coastal location, while NY2 was classified as a glaciated region based on its location in northwestern New York State. A spatial analysis of molecular variance (SAMOVA; Dupanloup et al., 2002) was used to characterize patterns of genetic structure across the species distribution. This approach operates under a simulated annealing procedure and iteratively seeks a user-defined number of groups (*K*) that maximizes the statistic *F*_CT_, which is an indicator of the proportion of total genetic variance due to differences between groups of populations, and minimizes *F*_SC_, the proportion of total genetic variance shared between spatial groups within groups. Each run began with a new random starting point, and 100 starting points (iterations) as recommended by the manual. For the SAMOVA analysis, 32 populations of *S. foetidus* with intra population samplings (*n* = 485 individuals) were analyzed. We repeated the analyses with different numbers of groups (*K* = 2 to 10 simulated groups) until the *F*_CT_ value (the proportion of genetic variation among groups) reached a plateau.

### Molecular dating and demographic analyses

Divergence times of eight haplotypes found in *S. foetidus* were estimated using Bayesian inference implemented in the software BEAST version 1.7.3 (Drummond and Rambaut, 2007). Genus *Symplocarpus* belongs to the subfamily Orontioideae, which is an early diverging lineage of Araceae, and the previous phylogenetic study (Nie et al., 2006) suggests the sister relationship between Orontioideae and Gymnostachydoideae. Thus, we used the same fossil calibration point as Nie et al. (2006) and the subfamily Orontioideae clade was constrained to 71 mya based on the fossil species of *Albertarum pueri*. We used *Gymnostachys* (the monotypic subfamily Gymnostachydoideae) as an outgroup (3 accessions) based on its sister relationship to the Orontioideae and included representative sequences of *Orontium* (4 accessions), *Lysichiton* (5 accessions for each species), *Symplocarpus nipponicus* (10 accessions), and *S. renifolius* (5 accessions from Japan). All these sequences were deposited in GenBank (accession numbers MH203650-MH203753). We used the GTR+I+G model, Yule process speciation prior, and lognormal relaxed clock. The analyses were run for 40 million generations, sampling every 5000 generations. Tracer v. 1.6.0 (Rambaut and Drummond, 2009) was used to estimate the posterior distribution of all statistics. All effective sample size (ESS) values were well above 200, which are considered to be a recommended threshold and indicate a stationary posterior distribution (Drummond and Rambaut, 2007). The program TreeAnnotator 1.7.3 (part of the BEAST 1.7.3 package) with 10% burn-in was applied to estimate mean divergence time and 95% highest posterior density (HPD) intervals. Finally, FigTree 1.3.1 (Rambaut, 2009) was used to display each node ages and their 95% HPD intervals.

For demographic history, mismatch distributions were calculated and tested against sudden demographic expansion (Rogers and Harpending, 1992) using Arlequin 3.5 (Excoffier and Lischer, 2010) and DnaSP v. 5 (Librado and Rozas, 2009). Unimodal patterns would be expected for recent sudden population expansions, while multimodal distributions are suggestive of demographic stability or multiple colonization (Slatkin and Hudson, 1991). Tajima's *D* (Tajima, 1989), Fu's *F*_*s*_ (Fu, 1997), and Ramos-Onsins and Rozas' *R*_2_ (Ramos-Onsins and Rozas, 2002) were also calculated to test for evidence of range expansion. To validate the fit of models, we used the sum of squared deviations (SSD; Schneider and Excoffier, 1999) between the observed and expected distribution and Harpending's raggedness index values (*RI*; Harpending, 1994) of the observed distribution. Significant positive Tajima's *D* and Fu's *F*_*s*_ values indicate no sudden expansion events.

### Ecological niche modeling

We performed environmental niche modeling (ENM) based on the current distribution of *S. foetidus* in eastern North America using present and past bioclimatic factors to predict putative species ranges during the last glacial maximum (LGM: 21,000 years before present) and the last inter-glacial (LIG: *c*. 130,000 years before present). We obtained the occurrences from the Global Biodiversity Information Facility (GBIF) database (http://www.gbif.org; accessed 14 November 2017) and our own field investigations. We manually filtered occurrences data, and sorted location inconsistences based on the expected distribution ranges. The final dataset included 1,524 occurrence records of *S. foetidus*. We used 19 bioclimatic factors as environmental data for ENM to predict the species' current distribution, then projected the species' probable distribution to the LGM and LIG. The environmental data describing the baseline climate (19 BioClim layers for the period 1,960–1,990 at a spatial resolution 2.5 arc-min, approximately 4.5 km^2^), data for the LGM with spatial resolution of 2.5 arc min resolution and for the LIG (Otto-Bliesner et al., 2006) at 30 arc sec resolution (exported to a resolution of 2.5 arc min for use in MaxEnt) were retrieved from the WorldClim version 1.4 data set (Hijmans et al., 2005). To reduce the effect of association between climate parameters, we calculated the correlation between all pairs of 19 parameters from the geographical points of the species occurrence using R (R Development Core Team, 2017). Although correlation tests found high correlation coefficients between several pairs of bioclimatic variables, we included all six variables because outcomes from models incorporating correlated variables are expected for prediction of current conditions and inclusion of correlated variables has been found to produce more accurate models for projections into past and future conditions (Braunisch et al., 2013). Six bioclimatic environmental variables include BIO1 (annual mean temperature), BIO6 (min temperature of coldest month), BIO11 (mean temperature of coldest quarter), BIO12 (annual precipitation), BIO13 (precipitation of wettest month), and BIO16 (precipitation of wettest quarter). We evaluated model performance using the data under the receiver operating characteristic curve (AUC) calculated by MaxEnt version 3.4.0 (Phillips et al., 2017). Our models had high discriminative power for the training data sets (AUC > 0.9673) and they were also able to predict the testing points (AUC > 0.9669). For the LGM and LIG model projections, we projected the model into Community Climate System Model (CCSM), downscaled to 2.5 arc-min (Hijmans et al., 2005). We performed 10 replicates for each analysis in the program MaxEnt. A total of 10,610 background points were randomly chosen for every simulation within the geographical area involved in each analysis. We used the default convergence threshold (10^−5^), maximum iterations (1,500) and 25% of the sites from model training. Habitat stability was calculated as the sum of logistic suitability values of niche projections during present, LGM, and LIG. Habit stability per population was calculated as the average of habitat stability values of each point location. Pearson correlation coefficients (*r*) were used to estimate the linear associations of habitat stability with haplotype diversity (Hd), nucleotide diversity (π), and private haplotype number. Furthermore, the associations of latitude with haplotype diversity, nucleotide diversity, and private haplotypes were estimated using R (R Development Core Team, 2017).

## Results

### Sequence variation

Four cpDNA non-coding regions (*psb*J*-pet*A: 1,128 bp; *trn*S*-trn*G: 995 bp; *rpl*32*-trn*L: 1,041 bp; *trn*Q*-5*′*rps*16: 877 bp) were sequenced from 485 individuals (32 populations) of *S. foetidus* in eastern North America. The aligned sequences were 4,041 bp long in total and contained 36 variable sites, including 8 nucleotide substitutions, 11 indels, and one inversion (17 bp inversion in *psb*J-*pet*A region). We excluded mononucleotide repeat regions and coded the remaining indels as binary character (A or T). We found one substitution (position 207) and one large inversion (positions 1,036–1,052) in *psb*J-*pet*A, two indels (positions 1,509–1,513 and 1,733–1,737) and one non-mononucleotide substitution/indel (position 1,738) in *trn*G-*trn*S, four substitutions (position 2,134, 2,257, 2,535, and 2,574) in *rpl*32-*trn*L, and three substitutions (position 3,445, 3,837, and 3,876) in *trn*Q-*rps*16 (Supplementary Table 1).

### Haplotype frequency and distribution

A total of eight chloroplast haplotypes (H1-H8) were identified from the data set. There were five haplotypes from the glaciated regions (H1-H3, H7, and H8), while eight haplotypes were found from the unglaciated regions (H1-H8) (Tables 1, 2, 4 and Figure 1). Five haplotypes were shared between the glaciated and the unglaciated regions: H1-H3, H7, and H8 (Table 2 and Supplementary Table 2). Three low frequency haplotypes, H4-H6, were found only in the unglaciated regions (PA1 and PA2). With the exception of two populations (CT1 and MA1), only one haplotype within a glaciated population was found. CT1 and MA1 populations were sampled from the east of the Appalachian Mountains and the boundary of southern limit of the Wisconsin glacier. Given the unknown precise southern range limit of the glacier, we cautiously categorized these two regions as the glaciated region. The population PA1 from the unglaciated region harbored the most number of within population haplotypes (H3-5 and H8), of which two were unique (Figure 1). Populations from the glaciated region contained a subset of haplotypes present in the unglaciated region and no unique haplotype was found from the glaciated region.

**Table 2:** Distribution of haplotypes in *Symplocarpus foetidus* among individuals, populations, and glaciated/unglaciated regions of eastern North America.

**Haplotype**	**Number of individuals**	**Frequencies in individuals(%)**	**Number of populations**	**Frequencies in populations(%)**	**Geographical distributions**	**Regional groups**
H1	196	40.4	12	37.5	IL1, IL2, IN1, IN2, MA1, MI2, OH1, WI1, WI2, *WV1, WV2,* *MD2*	Glaciated and *Unglaciated*
H2	60	12.3	5	15.6	*NC1, NJ1*, ON1, *TN1, VA3*	Glaciated and *Unglaciated*
H3	79	16.2	11	34.3	CT1, MA2, *MD1*, ME1, NB1, *NJ1, NJ2, NY1*, NY2, *PA1*, *WV2*	Glaciated and *Unglaciated*
H4	1	0.20	1	3.12	*PA1*	*Unglaciated*
H5	1	0.20	1	3.12	*PA1*	*Unglaciated*
H6	1	0.20	1	3.12	*PA2*	*Unglaciated*
H7	3	0.61	2	6.25	CT1, *NJ1*	Glaciated and *Unglaciated*
H8	144	29.6	12	37.5	*DE1*, MA1, *MD1*, MI1, *NC1, NJ2, NY1*, OH2, OH3, *PA1*, *PA2, VA2*	Glaciated and *Unglaciated*

Unglaciated populations are in italics. CT, Connecticut; DE, Delaware; IL, Illinois; IN, Indiana; MA, Massachusetts; MD, Maryland; ME, Maine; MI, Michigan; NB, New Brunswick; NC, North Carolina; NJ, New Jersey; NY, New York; OH, Ohio; ON, Ontario; PA, Pennsylvania; TN, Tennessee; VA, Virginia; WI, Wisconsin; WV, West Virginia

The H1 and H8 haplotypes were most common in *S. foetidus*, with a frequency of 40.4% (196 accessions) and 29.6% (144 accessions), respectively (Table 2). The haplotype H1 was found mostly in the glaciated Midwestern states (IL, IN, MI, OH, and WI) with the exception of three populations (WV1, WV2, and MD2) from the unglaciated region. The H8 haplotype was mostly found in the unglaciated region (DE, MD, NC, NJ, NY, PA, and VA) with just three from the glaciated region (OH, MA, and MI). The haplotype H2 was found in the unglaciated region (NC, NJ, TN, and VA) except for ON in the glaciated region (Table 2 and Figure 1). The H3 and H7 haplotypes were confined mostly to the northeastern regions of the North America (CT, MA, MD, NJ, ME, NY, PA, WV, and NB). Populations from the unglaciated region that were east of the Appalachian Mountains contained variable haplotypes compared to those from the glaciated region in the Midwest, which were west of the Appalachian Mountains. Two populations (CT1 and MA1) from the glaciated region contained two haplotypes; H3 and H7 in CT1 and H1 and H8 in MA1.

### Haplotype network

**Figure 2:**
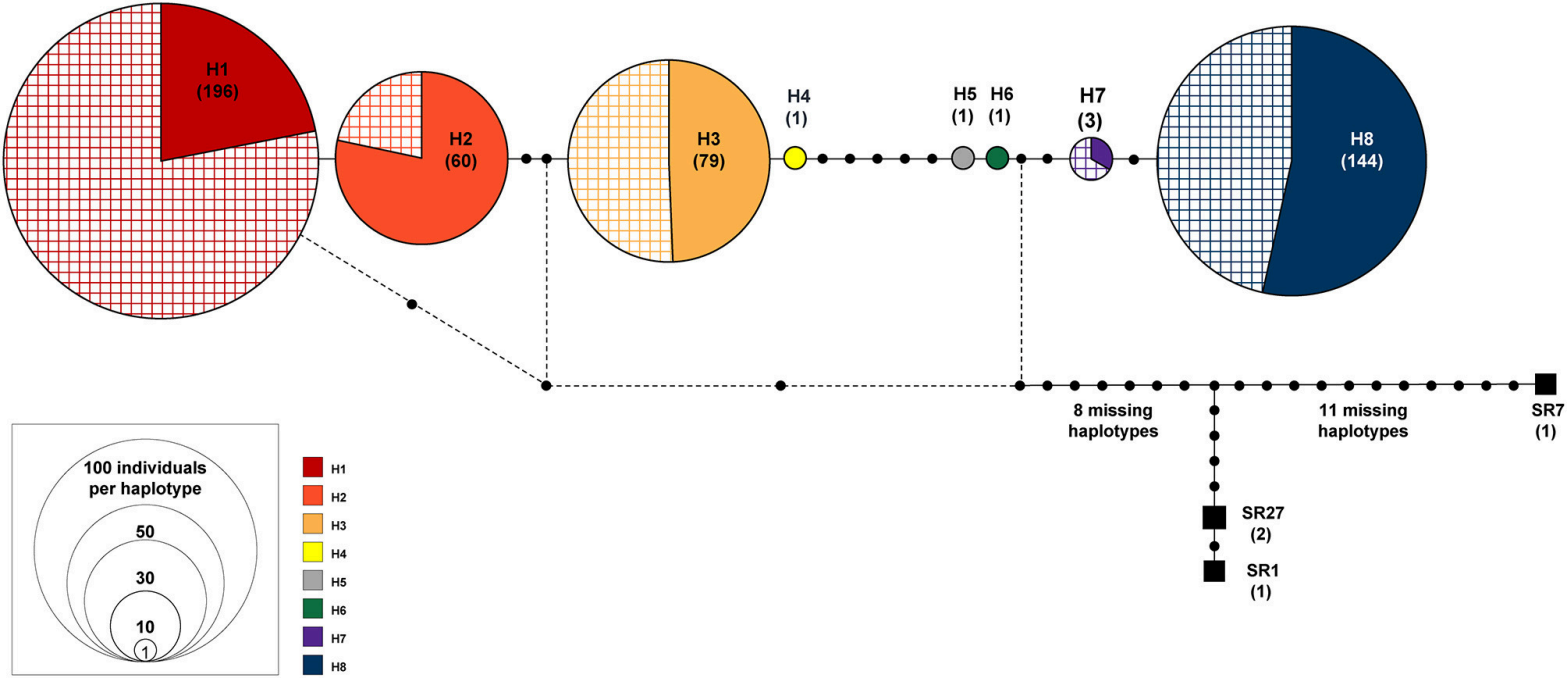
TCS haplotype network of eight haplotypes found in *Symplocarpus foetidus*. The size of each circle is proportional to relative haplotype frequency. The cross stripes patterned portion represents the individuals sampled from the glaciated region. Three haplotypes in black squares are *S. renifolius* from Japan (SR1, SR7, and SR27).

The genealogical relationships among haplotypes are nearly identical between the TCS and the median-joining network method and thus the haplotype network based on the TCS analysis is presented (Figure 2). Based on this network, two major groups could be recognized. The first group (haplogroup 1) included four haplotypes (H1, H2, H3, and H4). These haplotypes were found quite broadly from the unglaciated regions and east of the Appalachians (MD1, MD2, NC1, NJ1, NJ2, PA1, NY1, VA3, TN1, WV1, and WV2). This group was also represented in the glaciated states of the Midwest (ON1, WI1, WI2, IL1, IL2, IN1, IN2, OH1, and MI2) and northeastern regions (NB1, MA1, MA2, CT1, ME1, and NY2). The second group (haplogroup 2) included two low frequency haplotypes (H5 and H6) found only in Pennsylvania (PA1 and PA2) and two haplotypes (H7 and H8) from the glaciated (OH2, OH3, MI1, and MA1) and the unglaciated (DE1, MD1, NC1, NJ2, PA1, PA2, NY1, and VA2) regions (Supplementary Table 2).

### Phylogenetic analysis, molecular dating, and demographic analyses

**Figure 3:**
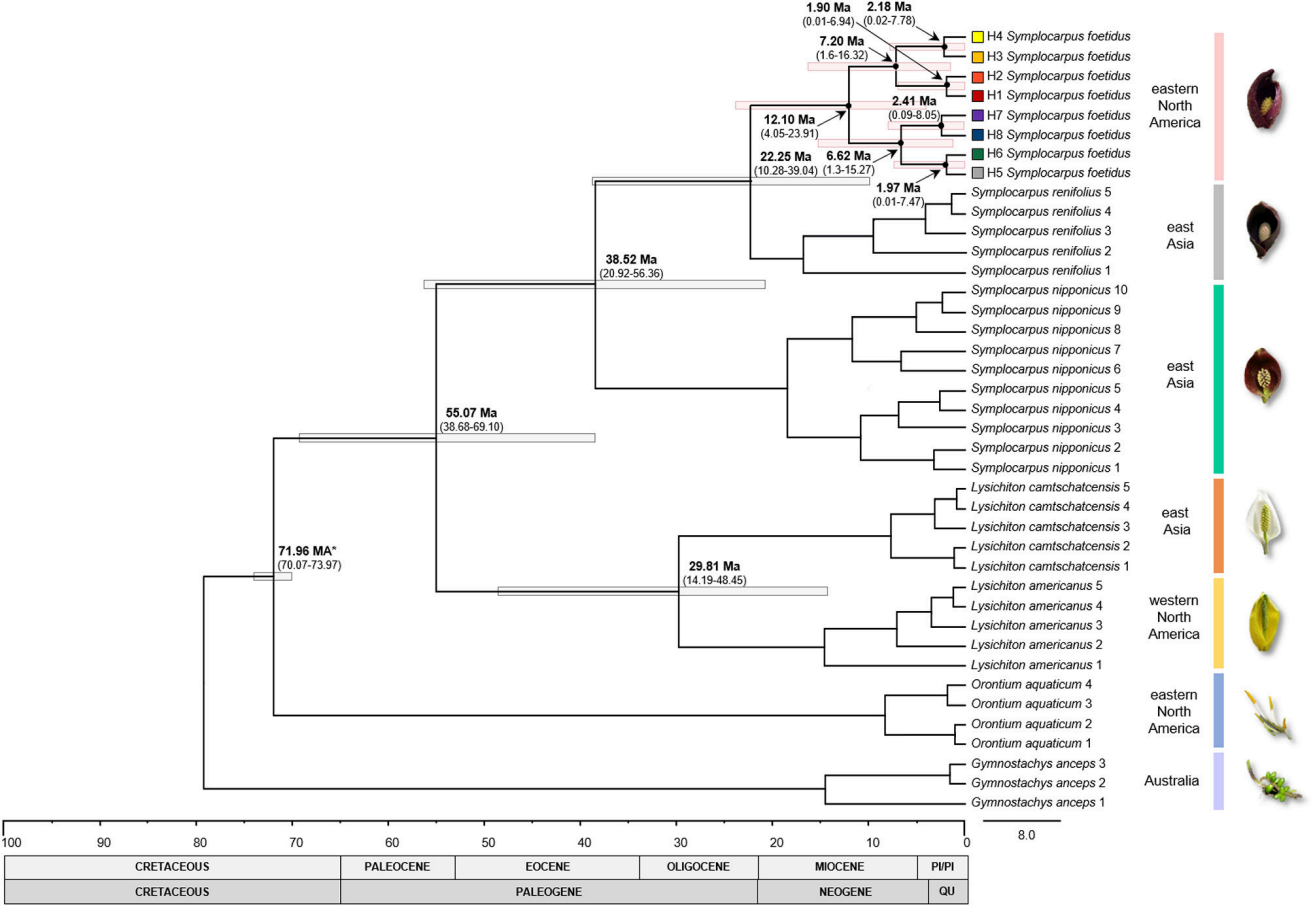
Chronogram of the subfamily Orontioideae constructed with BEAST. Calibrated node is indicated by the asterisk. Gray and pink bars indicate 95% HPD intervals for nodes of particular interest, with ages and 95% HPD given (in millions of years) above the bars. Haplotype numbers and color codes correspond to those in other Figures. PI/PI, Pliocene/Pleistocene; QU, Quaternary.

The ML analysis (not shown) and Bayesian dating (Figure 3) supported the same pattern observed in the network analysis. Two major lineages were identified with somewhat weak bootstrap support (BS) values. The major lineage 1 (68% BS) included the haplotypes H1-H4 and, with the exception of H4, which was found only in the unglaciated region, these haplotypes were found in both the unglaciated and glaciated regions. The major lineage 2 (83% BS) included the haplotypes H5-H8; H5 and H6 were limited to unglaciated regions, while H7 and H8 were found in both glaciated and unglaciated regions, although H7 rarely so. Bayesian dating suggested that the crown age of *S. foetidu*s haplotypes was estimated to be 12.10 mya (95% HPD, 4.05-23.91 mya) (Figure 3). Two intraspecific lineages within *S. foetidus* were estimated to be 7.20 mya (95% HPD, 1.6–16.32 mya) for the major lineage 1 and 6.62 mya (95% HPD, 1.3–15.27 mya) for the major lineage 2. Within each intraspecific lineage, haplotypes diverged during the Pleistocene, ranging from 1.90 mya (95% HPD, 0.01–6.94 mya) for H1 and H2 haplotypes to 2.41 mya (95% HPD, 0.09–8.05 mya) for H7 and H8 haplotypes. The divergence between *S. foetidus* in eastern North America and *S. renifolius* in East Asia was estimated to be 22. 25 mya (95% HPD, 10.28–39.04 mya).

**Figure 4:**
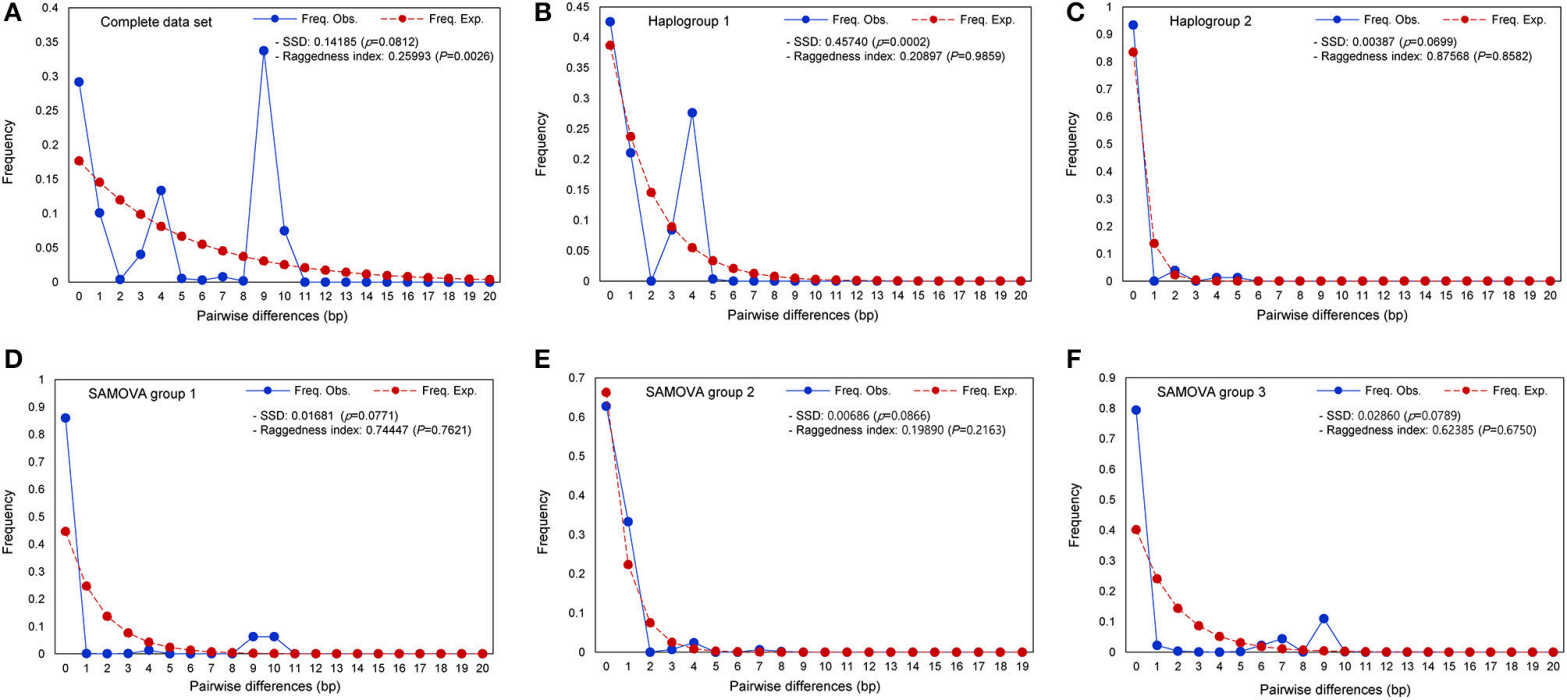
Pairwise haplotype mismatch distributions for populations of *S. foetidus*. Graphs show **(A)** complete data set, **(B)** haplogroup 1, **(C)** haplogroup 2, **(D)** SAMOVA group 1, **(E)** SAMOVA group 2, and **(F)** SAMOVA group 3.

Mismatch distributions of the complete data set of *S. foetidus* (32 populations and 485 individuals) were clearly multimodal (Figure 4A), suggesting constant population size, multiple colonization, and/or sustained subdivision for a long period of time. Mismatch distributions of haplogroup 1 were bimodal (Figure 4B), while dominant peak in frequency at one pairwise differences with much smaller peak at four pairwise differences was observed in haplogroup 2 (Figure 4C). In the three SAMOVA groups (Figures 4D–F), the pairwise mismatch distribution showed a dominant peak in frequency at 0 pairwise differences with a much smaller peak at 9-10 for SAMOVA group 1, 4 for SAMOVA group 2, and 9 for SAMOVA group 3, which was consistent with the inclusion of divergent haplotypes in the haplotype network (Figure 2). Tajima's *D*, Fu's *F*_*s*_, Ramos-Onsins and Rozas' *R*_2_, and the mismatch distribution failed to detect significant population expansion in *S. foetidus* (Table 3).

**Table 3:** Neutrality and population expansion tests for *Symplocarpus foetidus*.

**Groups**	**Tajima's *D* (*P*-value)**	**Fu's *Fs* (*P*-value)**	**Sum of squared deviation (SSD) (*P*-value)**	**Harpending's raggedness index (*RI*) (*P*-value)**	**Ramos-onsins and rozas (*R*_2_) (*P*-value)**
**COMPLETE DATA SET**					
32 populations 485 individuals	−0.05754^ns^ (0.57600)	−0.72691^ns^ (0.59800)	0.14185^ns^ (0.08120)	0.25993^*^ (0.00260)	0.06967^ns^ (1.00000)
**HAPLOGROUP**					
Haplogroup 1	−0.06894^ns^ (0.56700)	−0.31848^ns^ (0.55900)	0.45740^**^ (0.00020)	0.20897^ns^ (0.98590)	0.07715^ns^ (0.97700)
Haplogroup 2	−0.01666^ns^ (0.72000)	−0.07118^ns^ (0.49249)	0.00387^ns^ (0.06990)	0.87568^ns^ (0.85820)	0.09545^ns^ (0.10654)
Mean	−0.0428 (0.6435)	−0.19483 (0.525745)	0.23064^ns^ (0.03505)	0.54233^ns^ (0.92205)	0.0863^ns^ (0.54177)
**SAMOVA GROUP**					
SAMOVA group 1	−0.01676^ns^ (0.54400)	−0.10160^ns^ (0.54163)	0.01681^ns^ (0.07710)	0.74447^ns^ (0.76210)	0.08620^ns^ (0.26379)
SAMOVA group 2	0.00332^ns^ (0.57900)	−0.21136^ns^ (0.52059)	0.00686^ns^ (0.08660)	0.19890^ns^ (0.21630)	0.08199^ns^ (0.26203)
SAMOVA group 3	−0.09140^ns^ (0.58100)	−0.19409^ns^ (0.57874)	0.02860^ns^ (0.07890)	0.62385^ns^ (0.67500)	0.09774^ns^ (0.31864)
Mean	−0.03495^ns^ (0.56800)	−0.16901^ns^ (0.54698)	0.01742^ns^ (0.08087)	0.52241^ns^ (0.55113)	0.08864^ns^ (0.28148)

*Tajima's D, Fu's Fs statistic, Sum of Squared deviation (SSD), and Harpending's Raggedness index (RI), Ramos-Onsins and Rozas (R_2_) with significance derived from 10,000 simulations*.

* *Significant at P < 0.01*.

** *Significant at P < 0.001*.

ns, *Not significant*.

### Genetic diversity and population genetic structure

**Figure 5:**
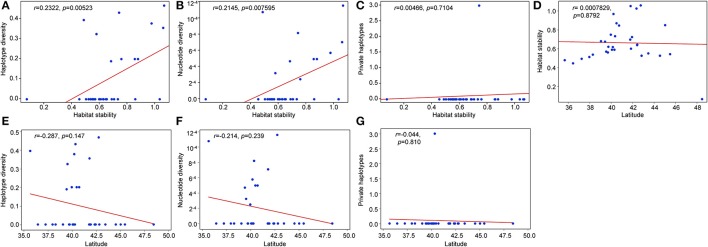
Pearson correlation coefficients between genetic diversity estimates and latitudinal location. **(A)** haplotype stability and haplotype diversity, **(B)** haplotype stability and nucleotide diversity, **(C)** habitat stability and private haplotypes, **(D)** latitude and habitat stability, **(E)** latitude and haplotype diversity, **(F)** latitude and nucleotide diversity, and **(G)** private haplotypes and latitude.

Nucleotide diversity (π) in the unglaciated region ranged from 0.0000 (DE1, MD2, TN1, VA2, VA3, and WV1) to 0.001079 (NC1) (Table 4). Haplotype diversity (Hd) ranged from 0.0000 (DE1, MD2, TN1, VA2, VA3, and WV1) to 0.4316 (PA1). For populations sampled from the glaciated region, nucleotide diversity was estimated to be 0.000707 (CT1), 0.001159 (MA1), and 0.0000 (the remaining populations). Haplotype diversity was estimated at 0.3556 (CT1), 0.4667 (MA1), and 0.0000 (the remaining populations). Overall, haplotype and nucleotide diversities were slightly higher in the unglaciated region (0.7425 ± 0.0122 and 0.001385 ± 0.000739, respectively) than the glaciated region (0.6099 ± 0.0230 and 0.001124 ± 0.000614, respectively). Pearson correlation coefficients showed that habitat stability was significantly and positively associated with all the genetic parameters (Figures 5A–C). Latitude, however, was not significantly associated with habitat stability or with genetic diversity estimates: latitude and habitat stability (*r* = 0.0007829, *p* = 0.8792), haplotype diversity and latitude (*r* = −0.287, *p* = 0.147), nucleotide diversity and latitude (*r* = −0.214, *p* = 0.239), and private haplotypes and latitude (*r* = −0.044, *p* = 0.810) (Figures 5D–G). In terms of the AMOVA results based on glaciated and unglaciated groupings, approximately 87% of total variation was explained by differences among populations within groups and ca. 15% due to differences within populations (Table 5). For three SAMOVA groupings, 87% of variation was partitioned among groups, and the remaining 3.4 and 9.5% among populations within groups and within populations, respectively. In the case of the SAMOVA analysis, we detected three phylogeographic groups (*F*_CT_ = 0.87115, *p* < 0.001) as the optimal number of genetic “groups” (*K*) based on spatial locations and cpDNA haplotypes. Although there was very little difference between three and four groups (*F*_CT_ = 0.88207, *p* < 0.001), *F*_CT_ (among-group variation) reached a plateau when the number of groups (*K*) was set at 3 (Supplementary Table 3 and Supplementary Figure 1). SAMOVA group 1 included the three western Appalachian populations (MI1, OH2, and OH3), one from the southern Appalachian Mountains (NC1), and five populations from the east coastal regions (DE1, MD1, PA2, MA1, and VA2) (Supplementary Table 4). The group 1 corresponded to the regions where haplotypes H1, H2, H3, H6, and H8 occurred. SAMOVA group 2 was one large grouping of 15 populations and 253 individuals that comprised multiple populations in all geographic regions: IL1, IL2, IN1, IN2, MI2, OH1, ON1, WI1, WI2, TN1, VA3, WV1, WV2, NJ1, and MD2. The second group was represented by H1, H2, H3, and H7. SAMOVA group 3 was exclusively located in northeastern states including NB1, ME1, MA2, CT1, NJ2, NY1, NY2, and PA1. This assemblage contained haplotypes H3, H4, H5, H7, and H8 in the TCS haplotype network (Figure 2). The three groups classified by SAMOVA analysis were similar to the groups identified in the haplotype networks and phylogenetic analysis. For example, two major haplogroups within *S. foetidus* were separated by five mutational steps, while two mutational steps separated each sub-haplogroup (Figure 2). Within each sub-haplogroup, one or two mutational step separate between haplotypes; two steps for between H7 and H8, and one step for between H1 and H2, between H3 and H4, and between H5 and H6. The sub-haplogroup containing H1 and H2 haplotypes contained mostly SAMOVA group 2 and very rarely SAMOVA group 1 (NC1 and MA1). The sub-haplogroup with H3 and H4 haplotypes contains mainly SAMOVA group 3 and less frequently SAMOVA group 2 (NJ1 and WV2) and SAMOVA group 1 (MD1). The second major clade (H5-H8) contained primarily SAMOVA group 1 and less frequently SAMOVA group 2 (NJ1) and SAMOVA group 3 (PA1, NJ2, CT1, and NY1).

**Table 4:** Summary of cpDNA variation for 32 populations of eastern North American *S. foetidus*.

**Populations**	**Sample size**	**No. of haplotypes**	**No. of polymorphic sites**	**Haplotype diversity (Hd)**	**Nucleotide diversity (π)**	**Tajima's** ***D***		**Fu's** ***F*****s**	
						***D***	***P*-value**	***F*s**	***P*-value**
**UNGLACIATED REGIONS (14 POPULATIONS WITH 210 INDIVIDUALS)**									
DE1	10	1	0	0.0000 ± 0.0000	0.0000 ± 0.0000	0.00000	1.00000	0.00000	N.A.
MD1	20	2	10	0.1895 ± 0.1081	0.00047 ± 0.000313	−1.12727	0.15000	5.11332	0.97200
MD2	10	1	0	0.0000 ± 0.0000	0.0000 ± 0.0000	0.00000	1.00000	0.00000	N.A.
NC1	20	2	11	0.3947 ± 0.1006	0.001079 ± 0.000622	1.40025	0.92100	9.64331	0.99900
NJ1	10	3	10	0.3778 ± 0.1813	0.000574 ± 0.000387	−1.47308	0.07000	2.35538	0.89200
NJ2	10	2	10	0.2000 ± 0.1541	0.000497 ± 0.000345	−1.90129	0.01000	3.98898	0.95900
NY1	10	2	10	0.2000 ± 0.1541	0.000497 ± 0.000345	−1.90129	0.00400	3.98898	0.94800
PA1	20	4	11	0.4316 ± 0.1262	0.000820 ± 0.000491	−0.07891	0.52600	3.57424	0.94600
PA2	10	2	5	0.2000 ± 0.1541	0.000248 ± 0.000206	−1.66706	0.03300	2.19722	0.82200
TN1	14	1	0	0.0000 ± 0.0000	0.0000 ± 0.0000	0.00000	1.00000	0.00000	N.A.
VA2	20	1	0	0.0000 ± 0.0000	0.0000 ± 0.0000	0.00000	1.00000	0.00000	N.A.
VA3	20	1	0	0.0000 ± 0.0000	0.0000 ± 0.0000	0.00000	1.00000	0.00000	N.A.
WV1	20	1	0	0.0000 ± 0.0000	0.0000 ± 0.0000	0.00000	1.00000	0.00000	N.A.
WV2	16	2	4	0.3250 ± 0.1251	0.000323 ± 0.000238	0.24535	0.64800	3.43030	0.93700
	210	8	13	0.7425 ± 0.0122	0.001385 ± 0.000739	3.26287	0.99900	10.05523	0.97600
**GLACIATED REGIONS (18 POPULATIONS WITH 275 INDIVIDUALS)**									
CT1	10	2	8	0.3556 ± 0.1591	0.000707 ± 0.000459	0.02480	0.53200	5.18801	0.98600
IL1	20	1	0	0.0000 ± 0.0000	0.0000 ± 0.0000	0.00000	1.00000	0.00000	N.A.
IL2	20	1	0	0.0000 ± 0.0000	0.0000 ± 0.0000	0.00000	1.00000	0.00000	N.A.
IN1	20	1	0	0.0000 ± 0.0000	0.0000 ± 0.0000	0.00000	1.00000	0.00000	N.A.
IN2	20	1	0	0.0000 ± 0.0000	0.0000 ± 0.0000	0.00000	1.00000	0.00000	N.A.
MA1	10	2	10	0.4667 ± 0.1318	0.001159 ± 0.000702	1.40202	0.93600	7.27204	0.99900
MA2	10	1	0	0.0000 ± 0.0000	0.0000 ± 0.0000	0.00000	1.00000	0.00000	N.A.
ME1	10	1	0	0.0000 ± 0.0000	0.0000 ± 0.0000	0.00000	1.00000	0.00000	N.A.
MI1	20	1	0	0.0000 ± 0.0000	0.0000 ± 0.0000	0.00000	1.00000	0.00000	N.A.
MI2	20	1	0	0.0000 ± 0.0000	0.0000 ± 0.0000	0.00000	1.00000	0.00000	N.A.
NY2	10	1	0	0.0000 ± 0.0000	0.0000 ± 0.0000	0.00000	1.00000	0.00000	N.A.
OH1	20	1	0	0.0000 ± 0.0000	0.0000 ± 0.0000	0.00000	1.00000	0.00000	N.A.
OH2	20	1	0	0.0000 ± 0.0000	0.0000 ± 0.0000	0.00000	1.00000	0.00000	N.A.
OH3	20	1	0	0.0000 ± 0.0000	0.0000 ± 0.0000	0.00000	1.00000	0.00000	N.A.
WI1	20	1	0	0.0000 ± 0.0000	0.0000 ± 0.0000	0.00000	1.00000	0.00000	N.A.
WI2	10	1	0	0.0000 ± 0.0000	0.0000 ± 0.0000	0.00000	1.00000	0.00000	N.A.
NB1	2	1	0	0.0000 ± 0.0000	0.0000 ± 0.0000	0.00000	1.00000	0.00000	N.A.
ON1	13	1	0	0.0000 ± 0.0000	0.0000 ± 0.0000	0.00000	1.00000	0.00000	N.A.
	275	5	12	0.6099 ± 0.0230	0.001124 ± 0.000614	3.07348	0.99800	13.25031	0.99000

*Hd, haplotype diversity; π, nucleotide diversity*.

**Table 5:** Result of the analysis of molecular variance (AMOVA) for 32 populations of *S. foetidus* using chloroplast DNA sequence data based upon geographical (glaciated vs. unglaciated) and SAMOVA groupings.

**Source of variation**	***d.f*.**	**SS**	**Variance components**	**Percentage of variation (%)**	**Fixation indices**	***P*-value**
**GROUPING 1: UNGLACIATED REGIONS (14 POPS) VS. GLACIATED REGIONS (18 POPS)**						
Among groups	1	28.377	−0.04291	−1.65	*F*_CT:_ −0.01653	0.43402 ± 0.01510
Among populations within groups	30	1035.591	2.27065	87.45	*F*_SC:_ 0.86029	0.00000 ± 0.00000
Within populations	453	167.050	0.36876	14.20	*F*_ST:_ 0.85798	0.00000 ± 0.00000
Total	484	1231.019	2.59650			
**GROUPING 2: SAMOVA GROUP 1 (9 POPS) VS. SAMOVA GROUP 2 (15 POPS) VS. SAMOVA GROUP 3 (8 POPS)**						
Among groups	2	995.687	3.38441	87.12	*F*_CT:_ 0.87115	0.00000 ± 0.00000
Among populations within groups	29	68.282	0.13181	3.39	*F*_SC:_ 0.26332	0.00000 ± 0.00000
Within populations	453	167.050	0.36876	9.49	*F*_ST:_ 0.90508	0.00000 ± 0.00000
Total	484	1231.019	3.88498			

*F_CT_: Proportion of genetic variation among groups*.

*F_SC_: Proportion of genetic variation between populations within groups*.

*F_ST_: Proportion of genetic variation between populations and groups overall*.

### Ecological niche modeling

**Figure 6:**
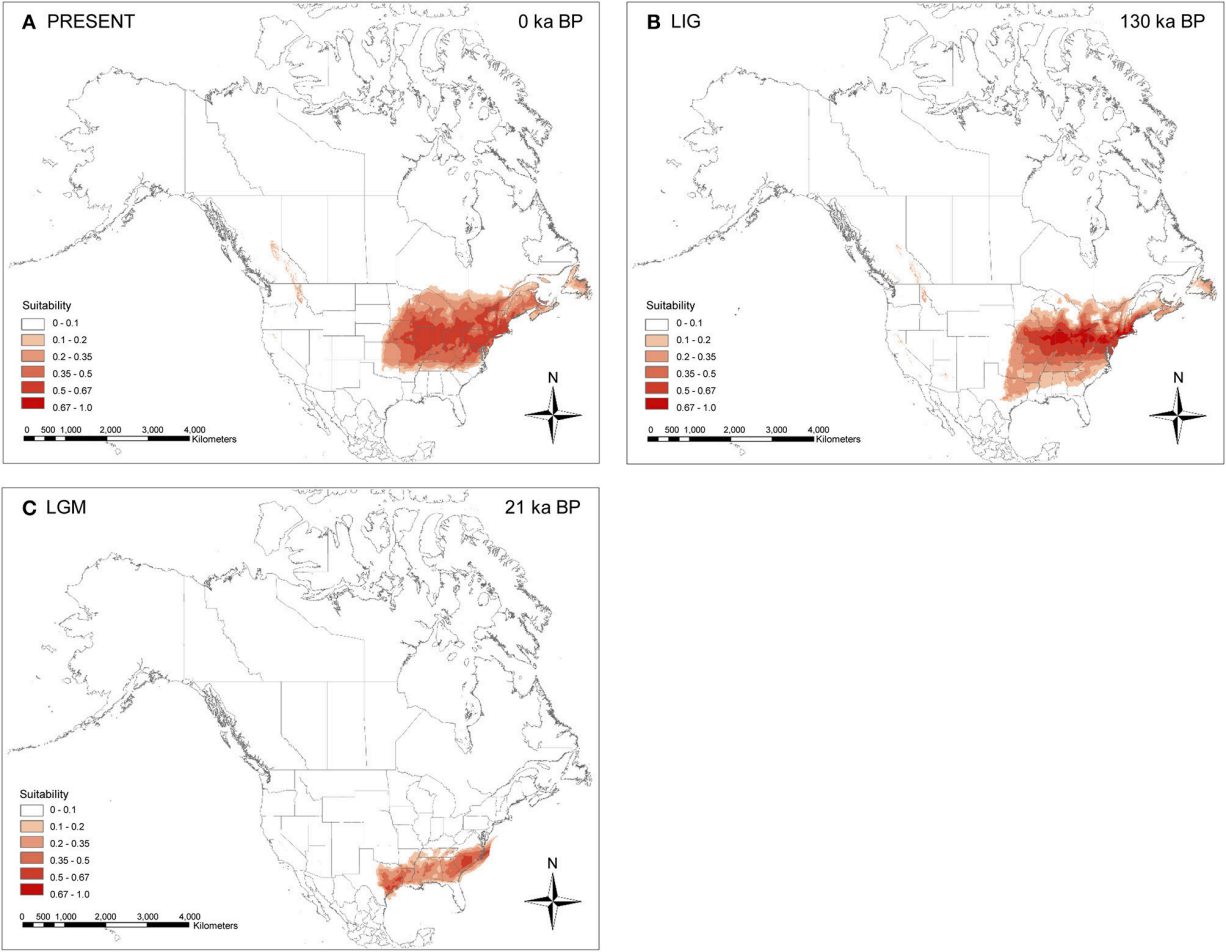
Map of ecological niche modeling of *Symplocarpus foetidus* using six climatic variables under the Community Climate System Model (CCSM). **(A)** Predicted distribution probability (in logistic value) for current climatic conditions. **(B)** Average projection of the model to the last interglacial [LIG: ca. 130 ka before present (BP)]. **(C)** Average projection of the model to the last glacial maximum (LGM: ca. 21 ka BP).

The cross validation of the climate envelope models revealed a high mean model fit with AUC = 0.9669 (SD 0.002). For the present (Figure 6A), these predicted areas usually included the species' known distribution in eastern North America. Further suitable habitat (with lower probability, <0.35) was predicted in the Rocky Mountains in British Columbia (Canada) and northwestern Idaho (United States), which are areas where the species is not known to occur. During the LIG (Figure 6B), the species' potential range was slightly larger, including several southern states (northeastern Texas, eastern Oklahoma, Arkansas, northern Mississippi, northern Alabama, northern Georgia, and northern South Carolina), and shifted farther south compared to the current distribution. During the LGM (Figure 6C), only southeastern states, from eastern Texas across the mid-Atlantic Coastal Plain and South Atlantic Coastal Plain, were predicted as suitable in eastern North America.

## Discussion

### Geographical patterns of genetic variation

Earlier allozyme studies reported reduced genetic variation in glaciated regions compared to regions that remained ice-free during the Wisconsin glaciation (Schwaegerle and Schaal, 1979; Lewis and Crawford, 1995; Broyles et al., 1997). In the case of one earlier phylogeographic study of *Trillium grandiflorum*, which has similar life history traits and distribution patterns as *S. foetidus*, no significant reduction in genetic diversity was reported between unglaciated and glaciated regions on the basis of allozyme diversity (Griffin and Barrett, 2004). *Trillium cuneatum*, a species that occurs in southeastern North America, survived the LGM in multiple refugia and migrated southwest to northeast through the Valley and Ridge regions of northwest Georgia (Gonzales et al., 2008). In the case of the herbaceous *Smilax herbacea* complex (Li et al., 2013), nucleotide diversity and haplotype diversity were slightly higher in Midwestern populations than in East Coast populations. In addition, the same number of haplotypes was found between the Midwest and the East Coastal regions. This pattern of genetic diversity was inferred to be the result of the existence of the “Driftless Area” as a Midwest northern refugium disjunct from far northwestern and southern refugia in the United States during the LGM (see discussion below). In our study, however, a reduction in haplotype number was evident from the unglaciated (8) to glaciated regions (5) in *S. foetidus* (Table 2). More importantly, all haplotypes found in the glaciated regions were also found in the unglaciated regions (Figure 2). Furthermore, we found a reduction in haplotype diversity and nucleotide diversity in the unglaciated (0.7425 ± 0.0122 and 0.001385 ± 0.000739) relative to the glaciated (0.6099 ± 0.0230 and 0.001124 ± 0.000614) regions. This lack of within-population diversity along with the patterns of haplotypes observed suggests that a northward, long distance seed dispersal from the glacial refugia. However, latitude was not significantly associated with any of the genetic diversity estimates (Figure 5). If populations expanded from the deep southern refugia (i.e., east and west of the southern Appalachians), we would expect to see a gradual decrease of genetic diversity toward the north (i.e., significant correlation between latitude and the genetic diversity estimates); however, this was not observed. In the case of *S. foetidus*, we suggest that present-day Pennsylvania, West Virginia, and New Jersey served as a “Northeastern refugium” rather than areas east and west of the southern refugia, in which there was no statistical significance of genetic diversity along the latitudinal gradient. Nevertheless, the overall pattern of genetic variation found in *S. foetidus* is consistent with less genetic variation above the glacial boundary than below it, a pattern documented in plants and animals (Comes and Kadereit, 1998; Avise, 2000; Hewitt, 2000; Soltis et al., 2006; Lee-Yaw et al., 2008; Russell et al., 2009).

### Glacial refugia and post-glacial range expansion

The “Driftless Area”, an unglaciated area encompassing present-day southwestern Wisconsin, southeastern Minnesota and northeastern Iowa, has been suggested as an important northern refugium for several plant and animal species during the LGM (Jackson and Overpeck, 2000; Holliday et al., 2002; Jaramillo-Correa et al., 2004; Rowe et al., 2004; Godbout et al., 2005; Lee-Yaw et al., 2008; Beatty and Provan, 2010; Li et al., 2013). Phylogeographical studies (e.g., Provan and Bennett, 2008), in general, suggest that high levels of genetic diversity and private haplotypes are expected in refugial areas. Given the similar modern-day geographic distributions and habitat in eastern North America between herbaceous *Smilax* and *Symplocarpus foetidus*, it is plausible that the “Driftless Area” served as a glacial refugium for *S. foetidus*; however, unique or endemic haplotypes in the “Driftless Area” were not observed in this study. Rather, one common haplotype (H1) was sampled from western Wisconsin and an adjacent Midwestern state (Illinois), and this area was most likely colonized by populations from the Appalachian Mountains [West Virginia; WV1, 654 m above sea level (a.s.l.) and WV2, 559 m a.s.l.] and east of the Appalachians (Maryland; MD2, 153 m a.s.l.). Because this same haplotype was shared among all but three populations (i.e., MI1, OH2, and OH3) from the Midwestern states (Wisconsin, Illinois, Ohio, and Michigan) this haplotype likely represents colonization by the populations from the Appalachians (Figure 1). Yet, we cannot rule out the possibility of this northern refugium, since we sampled only two populations from Wisconsin. Further study with comprehensive sampling from the presumably ice-free regions during the LGM, especially from southeastern Minnesota, northeastern Iowa, southwestern Wisconsin, and northwestern Illinois, may provide evidence for this refugium.

Of ten proposed glacial refugia for terrestrial plants and animals in northern North America based on previous phylogeographic studies (Figure 1 of Beatty and Provan, 2010), the data from *S. foetidus* appear to support the existence of a “Northeastern” refugium. Several phylogeographic studies suggested the importance of this putative eastern refugium for post-glacial recolonization of plants (e.g., *Dryas integrifolia*, Tremblay and Schoen, 1999; *Picea mariana*, Jaramillo-Correa et al., 2004; *Pinus banksiana*, Godbout et al., 2005) and animals (e.g., spring peeper *Pseudacris crucififer*, Austin et al., 2002; wood frog *Rana sylvatica*, Lee-Yaw et al., 2008). The populations sampled in the Appalachians (WV2) and east of the Appalachians (PA1 and NJ1) contained diverse haplotypes; some of which were shared between regions, while others were private. For example, the PA1 population (Pennsylvania) was sampled near the edge of the southeast glacier boundary and contained the most haplotype numbers (H3-5 and H8), private haplotypes (H4 and H5), and the highest haplotype (0.4316) and relatively high nucleotide (0.00082) diversity (Figure 1 and Table 4). One unique haplotype (H6) was also found in PA2 (Pennsylvania). Three haplotypes (H2, H3, and H7) were found from New Jersey (NJ1). Furthermore, all three geographic groups identified by SAMOVA are located in this region and high levels of genetic structure between geographically close populations in this region also suggest a putative northeastern refugium for *S. foetidus*. Therefore, this putative eastern refugium in present-day Pennsylvania, West Virginia, and New Jersey would have been close to the southernmost extent of the glacial ice and could have had a significant impact on recolonization to the glaciated regions in the west of the Appalachians as well as in northeastern North America (Holman, 1995; Lee-Yaw et al., 2008). The ecological niche modeling (Figure 6C), however, suggested that several states in the southernmost extent of the Wisconsin glacier ice, i.e., Pennsylvania, West Virginia, and New Jersey, were unsuitable habitats. The lack of suitable habitat in several states with high haplotype diversity, i.e., Pennsylvania, West Virginia, and New Jersey, may be due to resolution of the climatic datasets; resolutions are not fine enough to detect variation due to microclimates (Waltari et al., 2007; Gavin et al., 2014; Barnard-Kubow et al., 2015). It seems plausible that the topography of mountain ranges likely has produced fine-scale climatic variation and thus pocket refugia (e.g., sheltered valleys or south-facing slopes) farther north could not be detected via niche modeling (Waltari et al., 2007; Morris et al., 2008).

The data provided here suggest three possible northward post-glacial range expansions from the glacial refugia. Given the diversity of haplotypes in the SAMOVA group 1 and the identical haplotype (H8) found in the glaciated states (MI1, OH2 and OH3), this haplotype was recolonized by the east side Appalachian refugium located in Delaware, Maryland, Pennsylvania, and Virginia (Supplementary Figure 1). This refugium is also a likely source for northeastern route colonization found in glaciated region (MA1, Massachusetts). Haplotype H8 was commonly found in unglaciated northeastern regions, but it was also found in the southernmost population, NC1 (North Carolina) in this study. It is uncertain whether the disjunct distribution of this haplotype is due to the recent long dispersal of seeds or represents the relictual population once widely distributed in the Appalachian Mountains. Given the occurrence of two divergent haplotypes in Midwestern states (H1 and H8), it is highly likely that populations in eastern Michigan and Ohio (MI1, OH2, and OH3) were colonized independently from those in western part of Michigan and Ohio (OH1, MI2, IN1, IN2, IL1, IL2, WI1, and WI2). The SAMOVA group 2 includes the most populations sampled from the west side of Ohio and Michigan in the glaciated regions (i.e., Wisconsin, Illinois, western Michigan, Indiana, and western Ohio) and the populations sampled primarily from the Appalachian refugium found in Maryland, Virginia, Tennessee and West Virginia. The SAMOVA group 2 contains the common haplotype H1, which is most likely recolonized by the source population from West Virginia/Maryland. Haplotype H2, which occurs only in Ontario (the most northwestern population sampled in this study), also occurs in southern populations of the Appalachian Mountains (i.e., NC1 from North Carolina, TN1 from Tennessee, and VA3 from Virginia) (Figure 1). The sharing of the same haplotype H8 between Ontario, Canada and southern Appalachian states (including NJ1) was particularly intriguing since no intermediate populations between them had this haplotype (although sampling error cannot be ruled out completely). The SAMOVA group 3 (Supplementary Figure 1) includes the northeastern populations just below the boundary of ice-sheet found in Pennsylvania (PA1), New Jersey (NJ2) and New York (NY1) with the populations (i.e., CT1, MA2, ME1, NY2, and NB1) found above the glacier boundary mainly in the east of the Appalachian Mountains. This indicates a separate recolonization route from eastern refugium to primarily northeastern refugia.

Given that *S. foetidus* typically occurs in wetlands of eastern North America, this species likely shared its putative refugial areas with amphibians, and indeed reported amphibian refugia do share our predicted colonization routes for *S. foetidus* (Lee-Yaw et al., 2008 and references therein). For example, the refugium of the Northern Appalachian wood frog *Rana sylvatica* (“D” refugial area in Figure 6 of Lee-Yaw et al., 2008) is consistent with the refugium of *S. foetidus*. The northeastern colonization along the east side of the Appalachians and the western colonization toward the Midwestern states from the Northern Appalachian refugium (“D”) appear to be consistent with hypothesized colonization routes in *S. foetidus*. Another purported refugial area, the mid-Appalachians, the associated colonization routes of select amphibian species (*Plethodon crucifer*, Austin et al., 2002, 2004; *Ambystoma maculatum*, Zamudio and Savage, 2003; *Rana pipiens*, Hoffman and Blouin, 2004) are less consistent with this study because the H1 and H8 haplotypes were also found in the Northern Appalachian refugium. Two additional putative refugial areas of wood frogs, i.e., western Wisconsin refugium (“A”) and interior plains refugium in the American Midwest (“B”), were not consistent with predicted *S. foetidus* refugia. Further investigation is required to establish whether amphibian refugial areas and post-glacial colonization routes are shared with wetland plants in North America.

### Miocene origin

Our estimate of 12.10 mya (95% HPD, 4.06–23.91 mya) for the origin of *S. foetidus* in eastern North America is much older than the age estimate of the intercontinental split between East Asia (*S. renifolius*) and eastern North America (*S. foetidus*) within the genus *Symplocarpus* (ca. 6.88 ± 4.18 mya) (Nie et al., 2006). Our study also suggests that the two intraspecific lineages within *S. foetidus* originated during late Miocene. The fossil records suggested that the subfamily Orontioideae had a significantly broader geographic distribution during the Late Cretaceous and Paleogene throughout the Northern Hemisphere (Bogner et al., 2007; Kvaček and Smith, 2015). Specifically, within North America, which is where for the two subfamilies (Orontioideae and Gymnostachydoideae) are thought to have originated (Nauheimer et al., 2012), fossils of Orontioideae date to the Late Cretaceous and Eocene in climates ranging from warm subtropical to temperate, suggesting that the cool climatic tolerances of extant Orontioideae evolved around the Early Cenozoic (Bogner et al., 2007). Therefore, it is unlikely that our molecular dating overestimated the age of eastern North American *S. foetidus* even after considering several caveats associated with divergence time estimation (e.g., phylogenetic resolution, fossil position, mixed inter- and intraspecific data, gene tree instead of species tree, etc.) (Ho et al., 2005, 2007, 2008; Jennings and Edwards, 2005; Morris et al., 2008). The estimated divergence time of eastern North American *S. feotidus* appears to be congruent with other temperate woody taxa with similar geographic distribution (e.g., *Liquidambar styraciflua*, minimum of 8 myr for the divergence of two subclades within a species; Morris et al., 2008). Similar divergence times within the Altingiaceae of eastern North America and East Asia, ca. 22 mya (95% HPD, 9.74-44.59 mya), have also been estimated (see also Ickert-Bond and Wen, 2006; Morris et al., 2008). The results reported here for *S. foetidus* suggest that the modern geographic structure of eastern North American populations may be traced back to the Tertiary and that the inclusion of a temporal component in phylogeographic studies is critical in order to reflect dynamic biogeographical history (Dick et al., 2003; Magri et al., 2007; Morris et al., 2008; Bagnoli et al., 2015; Vitelli et al., 2017).

## Author contributions

S-HK and S-CK designed the experiment, drafted the manuscript. S-HK, M-SC, PL, and S-CK collected samples. S-HK performed the experiments. S-HK and M-SC analyzed the data. All of the authors have read and approved the final manuscript.

### Conflict of interest statement

The authors declare that the research was conducted in the absence of any commercial or financial relationships that could be construed as a potential conflict of interest.
