# Supplementary figures and images for: Phylogeography and Ecological Niche Modeling Reveal Reduced Genetic Diversity and Colonization Patterns of Skunk Cabbage (Symplocarpus foetidus; Araceae) From Glacial Refugia in Eastern North America
Source: Front Plant Sci. 2018 May 22;9:648. doi: 10.3389/fpls.2018.00648 (PMC5972301; doi:10.3389/fpls.2018.00648)

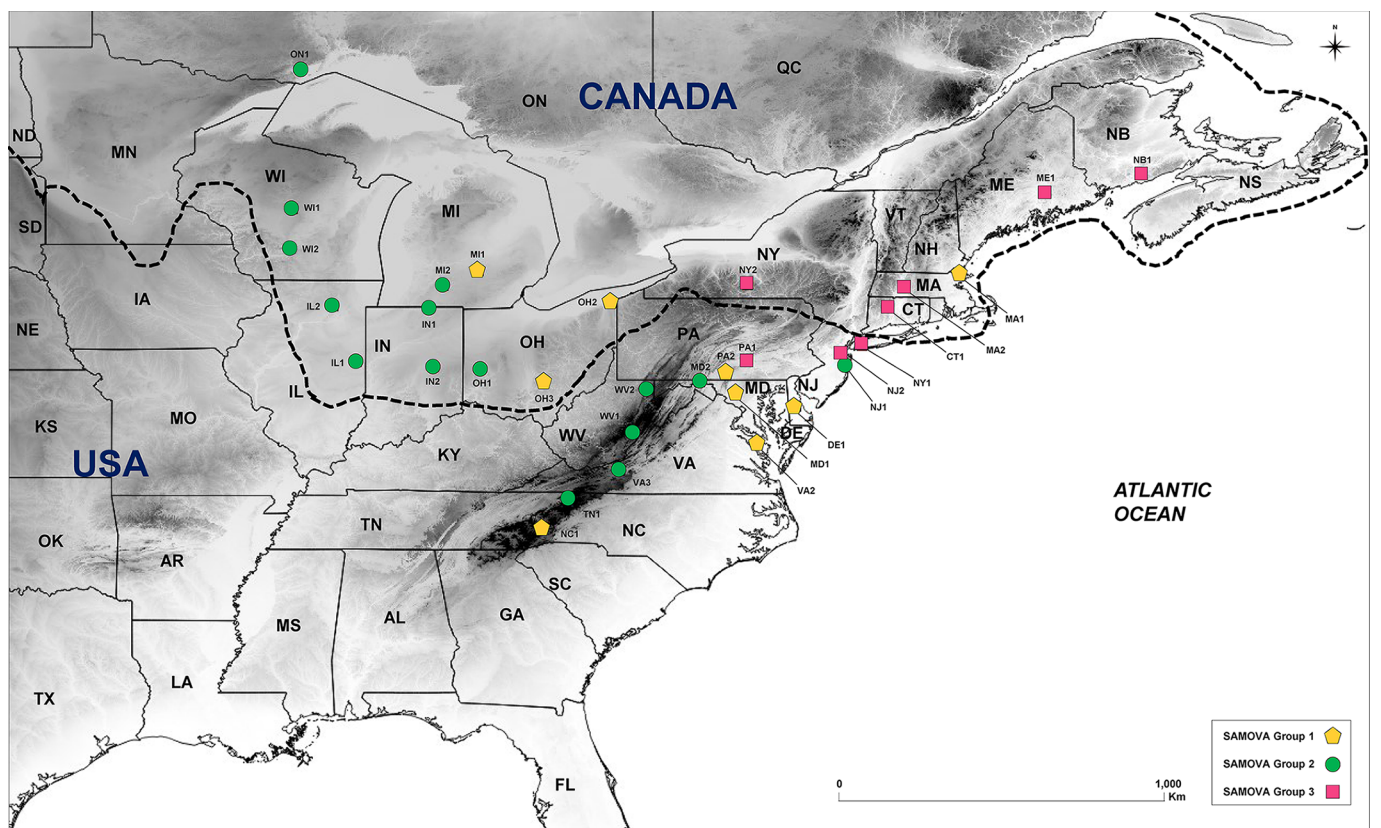

Supplement: Supplementary file 1 [file Image_1.pdf]
