# Supplementary material for: Phylogeography and Ecological Niche Modeling Reveal Reduced Genetic Diversity and Colonization Patterns of Skunk Cabbage (Symplocarpus foetidus; Araceae) From Glacial Refugia in Eastern North America
Source: Front Plant Sci. 2018 May 22;9:648. doi: 10.3389/fpls.2018.00648 (PMC5972301; doi:10.3389/fpls.2018.00648)
Supplement: Supplementary file 2 [file Table_1.docx]

Supplementary Material

Phylogeography and ecological niche modeling reveal reduced genetic diversity and colonization patterns of skunk cabbage (*Symplocarpus foetidus*; Araceae) from glacial refugium in eastern North America

Seon-Hee Kim, Myong-Suk Cho, Pan Li, and Seung-Chul Kim^*^

*** Correspondence**: Seung-Chul Kim: sonchus96@skku.edu

# Supplementary Figure and Tables

## 1.2 Supplementary Tables

**Supplementary Table 1**. Variable sites (substitutions, indels, and inversion) found in *Symplocarpus foetidus*, identifying 8 haplotypes (H1-H8).

| Haplotype | *psb*J*/pet*A  (1-1128 bp) | | | | | | | | | | | | | | | | | | *trn*G/S  (1129-2123 bp) | | | | | | | | | | | *rpl*32/*trn*L  (2124-3164 bp) | | | | *trn*Q/*rps*16 (3165-4041 bp) | | |
| --- | --- | --- | --- | --- | --- | --- | --- | --- | --- | --- | --- | --- | --- | --- | --- | --- | --- | --- | --- | --- | --- | --- | --- | --- | --- | --- | --- | --- | --- | --- | --- | --- | --- | --- | --- | --- |
|  | 207 | 1036 | 1037 | 1038 | 1039 | 1040 | 1041 | 1042 | 1043 | 1044 | 1045 | 1046 | 1047 | 1048 | 1049 | 1050 | 1051 | 1052 | 1509 | 1510 | 1511 | 1512 | 1513 | 1733 | 1734 | 1735 | 1736 | 1737 | 1738 | 2134 | 2257 | 2535 | 2574 | 3445 | 3837 | 3876 |
| H1 | G | C | T | C | A | A | A | T | A | G | A | C | A | A | A | A | C | A | T | A | T | T | A | T | A | T | A | T | T | A | G | T | G | T | C | T |
| H2 | G | T | G | T | T | T | T | G | T | C | T | A | T | T | T | G | A | G | T | A | T | T | A | T | A | T | A | T | T | A | G | T | G | T | C | T |
| H3 | G | T | G | T | T | T | T | G | T | C | T | A | T | T | T | G | A | G | T | A | T | T | A | T | A | T | A | T | T | A | T | T | A | T | C | G |
| H4 | G | T | G | T | T | T | T | G | T | C | T | A | T | T | T | G | A | G | T | A | T | T | A | T | A | T | A | T | T | C | T | T | A | T | C | G |
| H5 | G | C | T | C | A | A | A | T | A | G | A | C | A | A | A | A | C | A | - | - | - | - | - | T | A | T | A | T | A | C | G | T | A | G | T | G |
| H6 | G | C | T | C | A | A | A | T | A | G | A | C | A | A | A | A | C | A | - | - | - | - | - | T | A | T | A | T | A | A | G | T | A | G | T | G |
| H7 | T | C | T | C | A | A | A | T | A | G | A | C | A | A | A | A | C | A | - | - | - | - | - | - | - | - | - | - | - | A | G | T | A | G | T | G |
| H8 | T | C | T | C | A | A | A | T | A | G | A | C | A | A | A | A | C | A | - | - | - | - | - | - | - | - | - | - | - | A | G | A | A | G | T | G |

* Notes: One inversion event (1036-1052bp) was coded as A (TGTTTTGTCTATTTGAG) or T (CTCAAATAGACAAAACA).


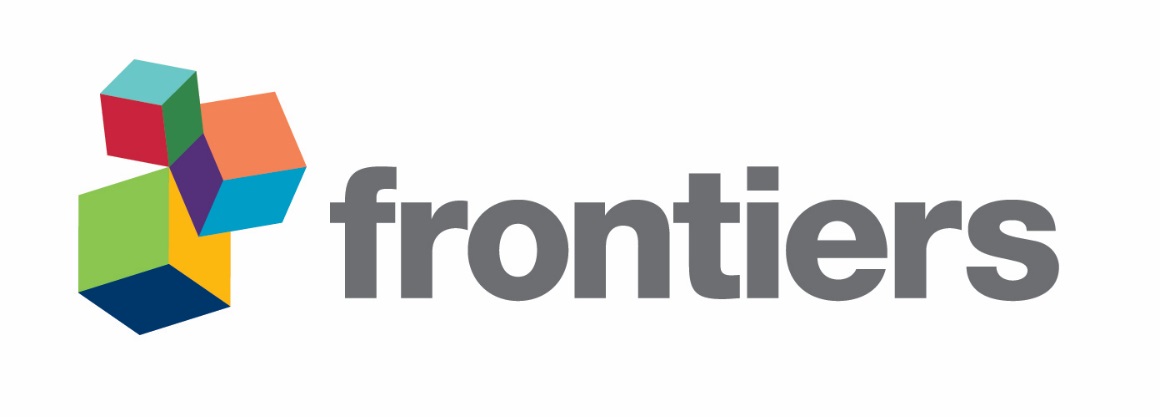


Supplementary Figure 1. The figure legends are required to have the same font as the main text, 12 point normal Times New Roman, single spaced. Please use a single paragraph for each legend and prepare the figures keeping in mind the PDF layout.
