# Supplementary material for: Phylogeography and Ecological Niche Modeling Reveal Reduced Genetic Diversity and Colonization Patterns of Skunk Cabbage (Symplocarpus foetidus; Araceae) From Glacial Refugia in Eastern North America
Source: Front Plant Sci. 2018 May 22;9:648. doi: 10.3389/fpls.2018.00648 (PMC5972301; doi:10.3389/fpls.2018.00648)
Supplement: Supplementary file 3 [file Table_2.docx]

Supplementary Material

Phylogeography and ecological niche modeling reveal reduced genetic diversity and colonization patterns of skunk cabbage (*Symplocarpus foetidus*; Araceae) from glacial refugium in eastern North America

Seon-Hee Kim, Myong-Suk Cho, Pan Li, and Seung-Chul Kim^*^

*** Correspondence**: Seung-Chul Kim: sonchus96@skku.edu

# Supplementary Figure and Tables

## Supplementary Tables

**Supplementary Table 2**. Haplotype frequencies found among populations of *Symplocarpus foetidus* in eastern North America.

|  | Unglaciated regions (14 populations with 210 individuals) | | | | | | | | | | | | | | Glaciated regions (18 populations with 275 individuals) | | | | | | | | | | | | | | | | | | Total |
| --- | --- | --- | --- | --- | --- | --- | --- | --- | --- | --- | --- | --- | --- | --- | --- | --- | --- | --- | --- | --- | --- | --- | --- | --- | --- | --- | --- | --- | --- | --- | --- | --- | --- |
|  | DE1 | MD1 | MD2 | NC1 | NJ1 | NJ2 | PA1 | PA2 | NY1 | VA2 | VA3 | TN1 | WV1 | WV2 | ON1 | WI1 | WI2 | IL1 | IL2 | IN1 | IN2 | OH1 | OH2 | OH3 | MI1 | MI2 | NB1 | MA1 | MA2 | CT1 | ME1 | NY2 |  |
|  | 10 | 20 | 10 | 20 | 10 | 10 | 20 | 10 | 10 | 20 | 20 | 14 | 20 | 16 | 13 | 20 | 10 | 20 | 20 | 20 | 20 | 20 | 20 | 20 | 20 | 20 | 2 | 10 | 10 | 10 | 10 | 10 |  |
| H1 | 0 | 0 | 10 | 0 | 0 | 0 | 0 | 0 | 0 | 0 | 0 | 0 | 20 | 13 | 0 | 20 | 10 | 20 | 20 | 20 | 20 | 20 | 0 | 0 | 0 | 20 | 0 | 3 | 0 | 0 | 0 | 0 | 196 |
| H2 | 0 | 0 | 0 | 5 | 8 | 0 | 0 | 0 | 0 | 0 | 20 | 14 | 0 | 0 | 13 | 0 | 0 | 0 | 0 | 0 | 0 | 0 | 0 | 0 | 0 | 0 | 0 | 0 | 0 | 0 | 0 | 0 | 60 |
| H3 | 0 | 2 | 0 | 0 | 1 | 9 | 15 | 0 | 9 | 0 | 0 | 0 | 0 | 3 | 0 | 0 | 0 | 0 | 0 | 0 | 0 | 0 | 0 | 0 | 0 | 0 | 2 | 0 | 10 | 8 | 10 | 10 | 79 |
| H4 | 0 | 0 | 0 | 0 | 0 | 0 | 1 | 0 | 0 | 0 | 0 | 0 | 0 | 0 | 0 | 0 | 0 | 0 | 0 | 0 | 0 | 0 | 0 | 0 | 0 | 0 | 0 | 0 | 0 | 0 | 0 | 0 | 1 |
| H5 | 0 | 0 | 0 | 0 | 0 | 0 | 1 | 0 | 0 | 0 | 0 | 0 | 0 | 0 | 0 | 0 | 0 | 0 | 0 | 0 | 0 | 0 | 0 | 0 | 0 | 0 | 0 | 0 | 0 | 0 | 0 | 0 | 1 |
| H6 | 0 | 0 | 0 | 0 | 0 | 0 | 0 | 1 | 0 | 0 | 0 | 0 | 0 | 0 | 0 | 0 | 0 | 0 | 0 | 0 | 0 | 0 | 0 | 0 | 0 | 0 | 0 | 0 | 0 | 0 | 0 | 0 | 1 |
| H7 | 0 | 0 | 0 | 0 | 1 | 0 | 0 | 0 | 0 | 0 | 0 | 0 | 0 | 0 | 0 | 0 | 0 | 0 | 0 | 0 | 0 | 0 | 0 | 0 | 0 | 0 | 0 | 0 | 0 | 2 | 0 | 0 | 3 |
| H8 | 10 | 18 | 0 | 15 | 0 | 1 | 3 | 9 | 1 | 20 | 0 | 0 | 0 | 0 | 0 | 0 | 0 | 0 | 0 | 0 | 0 | 0 | 20 | 20 | 20 | 0 | 0 | 7 | 0 | 0 | 0 | 0 | 144 |

*Abbreviations of populations are as follows: CT, Connecticut; DE, Delaware; IL, Illinois; IN, Indiana; MA, Massachusetts; MD, Maryland; ME, Maine; MI, Michigan; NB, New Brunswick; NC, North Carolina; NJ, New Jersey; NY, New York; OH, Ohio; ON, Ontario; PA, Pennsylvania; TN, Tennessee; VA, Virginia; WI, Wisconsin; WV, West Virginia


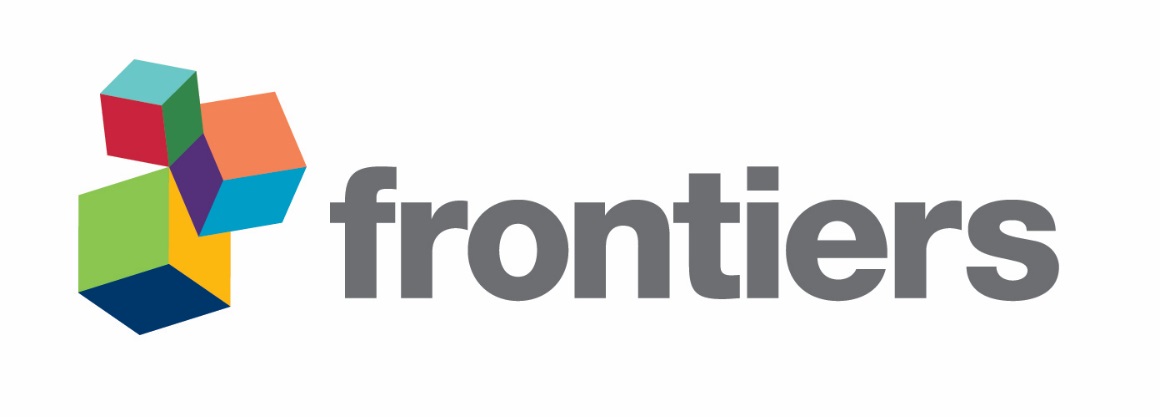


Supplementary Figure 1. The figure legends are required to have the same font as the main text, 12 point normal Times New Roman, single spaced. Please use a single paragraph for each legend and prepare the figures keeping in mind the PDF layout.
