# Supplementary material for: Phylogeography and Ecological Niche Modeling Reveal Reduced Genetic Diversity and Colonization Patterns of Skunk Cabbage (Symplocarpus foetidus; Araceae) From Glacial Refugia in Eastern North America
Source: Front Plant Sci. 2018 May 22;9:648. doi: 10.3389/fpls.2018.00648 (PMC5972301; doi:10.3389/fpls.2018.00648)
Supplement: Supplementary file 4 [file Table_3.docx]

Supplementary Material

Phylogeography and ecological niche modeling reveal reduced genetic diversity and colonization patterns of skunk cabbage (*Symplocarpus foetidus*; Araceae) from glacial refugium in eastern North America

Seon-Hee Kim, Myong-Suk Cho, Pan Li, and Seung-Chul Kim^*^

*** Correspondence**: Seung-Chul Kim: sonchus96@skku.edu

# Supplementary Figure and Tables

## Supplementary Tables

**Supplementary Table 3**. Comparison of fixation indices corresponding to the groups of populations detected by spatial analysis of molecular variance (SAMOVA) in *Symplocarpus foetidus* in USA and Canada based on four chloroplast DNA regions.

| No. of  groups (*K*) | Percentage of variation | | | Fixation indices | | |
| --- | --- | --- | --- | --- | --- | --- |
|  | Among  groups | Among populations  within groups | Within populations | *F*_SC_ | *F*_ST_ | *F*_CT_ |
| 2 | 80.20 | 12.01 | 7.79 | 0.60652 | 0.92208 | 0.80198 |
| 3 | 87.12 | 3.39 | 9.49 | 0.26332 | 0.90508 | 0.87115 |
| 4 | 88.21 | 1.14 | 10.66 | 0.09627 | 0.89342 | 0.88207 |
| 5 | 88.87 | 0.10 | 11.03 | 0.00922 | 0.88971 | 0.88868 |
| 6 | 88.98 | -0.18 | 11.20 | -0.01667 | 0.88798 | 0.88982 |
| 7 | 89.01 | -0.27 | 11.25 | -0.02420 | 0.88747 | 0.89013 |
| 8 | 89.02 | -0.29 | 11.27 | -0.02676 | 0.88725 | 0.89019 |
| 9 | 89.01 | -0.35 | 11.34 | -0.03166 | 0.88659 | 0.89007 |
| 10 | 88.92 | -0.48 | 11.56 | -0.04364 | 0.88435 | 0.88919 |

*F*_SC_: Proportion of genetic variation between populations within groups

*F*_ST_: Proportion of genetic variation between populations and groups overall

*F*_CT_: Proportion of genetic variation among groups


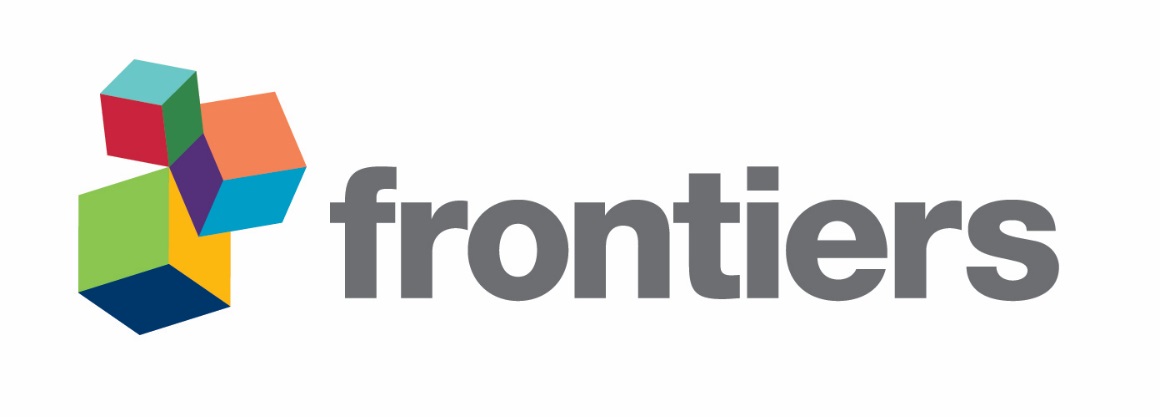


Supplementary Figure 1. The figure legends are required to have the same font as the main text, 12 point normal Times New Roman, single spaced. Please use a single paragraph for each legend and prepare the figures keeping in mind the PDF layout.
