# Supplementary material for: Phylogeography and Ecological Niche Modeling Reveal Reduced Genetic Diversity and Colonization Patterns of Skunk Cabbage (Symplocarpus foetidus; Araceae) From Glacial Refugia in Eastern North America
Source: Front Plant Sci. 2018 May 22;9:648. doi: 10.3389/fpls.2018.00648 (PMC5972301; doi:10.3389/fpls.2018.00648)
Supplement: Supplementary file 5 [file Table_4.docx]

Supplementary Material

Phylogeography and ecological niche modeling reveal reduced genetic diversity and colonization patterns of skunk cabbage (*Symplocarpus foetidus*; Araceae) from glacial refugium in eastern North America

Seon-Hee Kim, Myong-Suk Cho, Pan Li, and Seung-Chul Kim^*^

*** Correspondence**: Seung-Chul Kim: sonchus96@skku.edu

# Supplementary Figure and Tables

## Supplementary Tables

**Supplementary Table 4**. Genetic structure in *Symplocarpus foetidus*. The SAMOVA detected three phylogeographic groups (*F*_CT_ = 0.87115, p < 0.001) based on spatial locations and cpDNA haplotypes.

|  | Number of Populations | Number of Individuals | Glaciated  populations | Unglaciated  populations | Haplotypes |
| --- | --- | --- | --- | --- | --- |
| SAMOVA  group 1 | 9 | 150 | MA1  MI1  OH2  OH3 | DE1  MD1  NC1  PA2  VA2 | H1 (3)  H2 (5)  H3 (2)  H6 (1)  H8 (139) |
| SAMOVA  group 2 | 15 | 253 | IL1  IL2  IN1  IN2  MI2  OH1  ON1  WI1  WI2 | MD2  NJ1  TN1  VA3  WV1  WV2 | H1 (193)  H2 (55)  H3 (4)  H7 (1) |
| SAMOVA  group 3 | 8 | 82 | CT1  MA2  ME1  NB1  NY2 | NJ2  NY1  PA1 | H3 (73)  H4 (1)  H5 (1)  H7 (2)  H8 (5) |
|  | 32  populations | 485  individuals | 18 | 14 | 485 |

*Abbreviations of populations are as follows: CT, Connecticut; DE, Delaware; IL, Illinois; IN, Indiana; MA, Massachusetts; MD, Maryland; ME, Maine; MI, Michigan; NB, New Brunswick; NC, North Carolina; NJ, New Jersey; NY, New York; OH, Ohio; ON, Ontario; PA, Pennsylvania; TN, Tennessee; VA, Virginia; WI, Wisconsin; WV, West Virginia


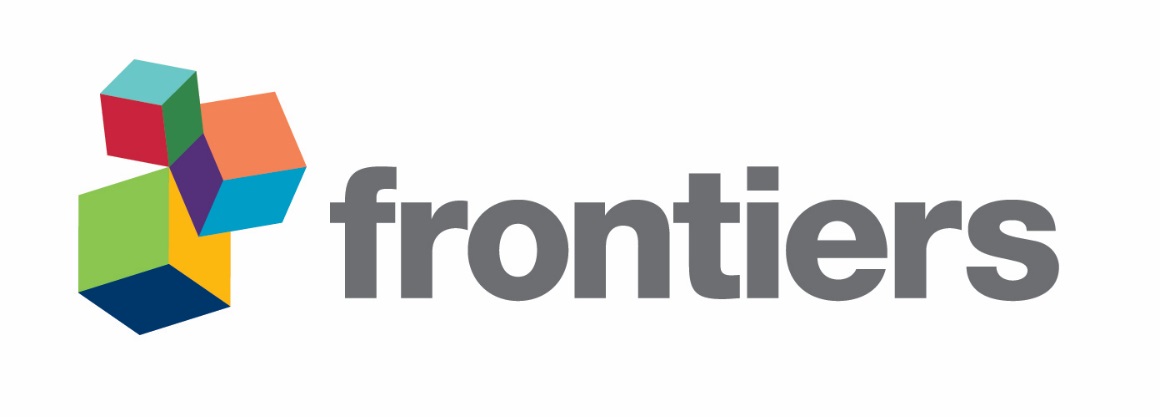


Supplementary Figure 1. The figure legends are required to have the same font as the main text, 12 point normal Times New Roman, single spaced. Please use a single paragraph for each legend and prepare the figures keeping in mind the PDF layout.
